# Three-dimensional cardiac computational modelling: methods, features and applications

**DOI:** 10.1186/s12938-015-0033-5

**Published:** 2015-04-17

**Authors:** Alejandro Lopez-Perez, Rafael Sebastian, Jose M Ferrero

**Affiliations:** Centre for Research and Innovation in Bioengineering (Ci2B), Universitat Politècnica de València, València, Spain; Computational Multiscale Physiology Lab (CoMMLab), Universitat de València, València, Spain

**Keywords:** Cardiac modelling, Three-dimensional (3D) modelling, Computational modelling, Fibre orientation, Cardiac conduction system (CCS), Cardiac image segmentation, Biophysical simulation, Personalisation, Patient-specific modelling

## Abstract

The combination of computational models and biophysical simulations can help to interpret an array of experimental data and contribute to the understanding, diagnosis and treatment of complex diseases such as cardiac arrhythmias. For this reason, three-dimensional (3D) cardiac computational modelling is currently a rising field of research. The advance of medical imaging technology over the last decades has allowed the evolution from generic to *patient-specific* 3D cardiac models that faithfully represent the anatomy and different cardiac features of a given alive subject. Here we analyse sixty representative 3D cardiac computational models developed and published during the last fifty years, describing their information sources, features, development methods and online availability. This paper also reviews the necessary components to build a 3D computational model of the heart aimed at biophysical simulation, paying especial attention to cardiac electrophysiology (EP), and the existing approaches to incorporate those components. We assess the challenges associated to the different steps of the building process, from the processing of raw clinical or biological data to the final application, including image segmentation, inclusion of substructures and meshing among others. We briefly outline the personalisation approaches that are currently available in 3D cardiac computational modelling. Finally, we present examples of several specific applications, mainly related to cardiac EP simulation and model-based image analysis, showing the potential usefulness of 3D cardiac computational modelling into clinical environments as a tool to aid in the prevention, diagnosis and treatment of cardiac diseases.

**Electronic supplementary material:**

The online version of this article (doi:10.1186/s12938-015-0033-5) contains supplementary material, which is available to authorized users.

## Introduction

Some decades ago, three-dimensional (3D) cardiac computational models were only used for very simple computational simulations of cardiac electrophysiology (EP) or cardiac mechanics analysis. Nowadays, 3D cardiac models are becoming increasingly complex and are currently used in other areas such as cardiac image segmentation, statistical modelling of cardiac anatomy, patient risk stratification or surgical planning. These models are starting to be used in clinical environments for 3D image analysis or therapy guidance in procedures such as radiofrequency ablation (RFA). Due to the intensive research in this field and the evolution of computing resources, the introduction of 3D advanced computational simulations of cardiac EP and/or mechanics and model-based cardiac image analysis in clinical environments are becoming more feasible.

This paper presents a review of the methods used to construct 3D cardiac computational models since their earliest developments (about fifty years ago) until today, and discusses their advantages and applicability to different areas. To carry out our systematic review, sixty representative computational models were taken from the literature and were analysed in order to explore the evolution of the methods used to develop 3D cardiac models over the last fifty years. As a main result, we crafted a wide summary table (see Additional file 1: Table S1) that provides information about the main features of the reviewed 3D cardiac models and the particular methods used to build each of them.

This article is organised as follows. We first discuss the information contained in (Additional file 1: Table S1) and its intended usefulness for the readers. Later, we outline the evolution of 3D cardiac models from the “early era” to the present days, highlighting the methods used for the computational reconstruction of cardiac anatomy. The next section addresses the different stages of the development process of a 3D cardiac model (3D reconstruction of cardiac anatomy, meshing, etc.) and reviews the available methods to construct a model and to include certain heart features (fibre orientation, cardiac conduction system, ischaemic scars, etc.) in a computational model aimed at biophysical simulation with especial attention to cardiac EP. The following section briefly describes the available personalisation approaches in cardiac computational modelling. Finally, the paper addresses the main applications of 3D cardiac models by presenting examples related to several specific applications, focusing on cardiac EP simulation and model-based image segmentation.

## Table of 3D cardiac computational models

Reviewing the entire literature related to the development of 3D cardiac models would be virtually impossible. For this reason, we chose sixty models from the literature as a representative set suitable to outline the evolution of 3D cardiac computational modelling from its beginning. In order to show this evolution we list them in chronological order in (see Additional file 1: Table S1).

Additional file 1: Table S1, crafted as a main result of this review work, was designed to provide a complete summary about the reviewed models. It shows information about the data source and methods used to develop each of the sixty reviewed 3D cardiac models as well as their main features, final application and online availability, in case the reader is interested in downloading any of them. The information provided by each column of Additional file 1: Table S1 is later addressed in a specific subsection of the *Elements of a 3D cardiac computational model* section, discussing why certain features or methods are needed or convenient for particular applications. We intend for readers to use Additional file 1: Table S1 as a reference tool along the entire article since it contains examples of models including the different cardiac features addressed or models that were developed using some of the methods mentioned in the text. Therefore, it can be used to find several models sharing a particular purpose or certain feature/method in which the reader might be especially interested or to compare different models in a quick and straightforward manner.

## Evolution of 3D models of cardiac anatomy

The first step of the development process of a 3D cardiac model is the computational reconstruction of the anatomy of the heart by generating a 3D cardiac geometry. In this section, a brief survey of the evolution of 3D cardiac models is presented focusing on the methods used to build the computational reconstruction of cardiac anatomy and the achieved level of anatomical detail.

### Generic models

The first developed 3D computational models of cardiac anatomy were simplistic models based on *geometric shapes*. Most of them only included the left ventricle (LV), represented by two concentric ellipsoids truncated at the base level to roughly approximate the shape of the LV [1-5]. However, this approach is still in use for specific applications in which the anatomical realism is not crucial for the purpose of the model [6,7].

Later, *anatomical models* were established. They aimed to represent cardiac anatomy in a more realistic fashion but still with a low level of anatomical detail due to the poor quality of the data used to build them. They were usually constructed by manual drawing from histo-anatomical slices [8-11] or from measurements taken on explanted hearts [12,13] or by segmenting pictures of histo-anatomical slices [14-17]. The most representative ones are two bi-ventricular models highly referenced and reused: the rabbit model from University of California San Diego [11] and the canine model from University of Auckland [12]. Their main contribution was the inclusion of realistic fibre orientation obtained from experimental measurements.

The development of computer-aided design (CAD) tools enabled the construction of 3D cardiac models without any direct source of anatomical information [18-21]. Some anatomical details, such as chambers volumes or wall thickness were just taken from the literature in order to virtually generate the geometry of the model.

3D *atrial models* began proliferating later than ventricular ones for several reasons, such as the higher lethality of ventricular disorders or the challenges associated to its 3D reconstruction due to the high complexity and inter-subject variability of atrial anatomy. Nevertheless, all kinds of model described above are present among reviewed 3D atrial models: geometric models [22], CAD models [19,21] and anatomical models from histo-anatomical slices [23-25].

### Medical image-based models

The evolution of medical imaging technology gave the possibility of building realistic 3D cardiac models from either *in-vivo* or *ex-vivo* images, as demonstrated by early works [26] and [27], respectively. *Medical image-based 3D cardiac models* have proliferated over the last 15 years due to the advance and consolidation of techniques such as magnetic resonance imaging (MRI) [28-34] and computed tomography (CT) [35,36], leading to the rise of 3D cardiac computational modelling. As will be discussed below, the development of new imaging modalities capable of providing structural and functional information of cardiac tissue was also a major breakthrough in 3D cardiac computational modelling.

**Figure 1:**
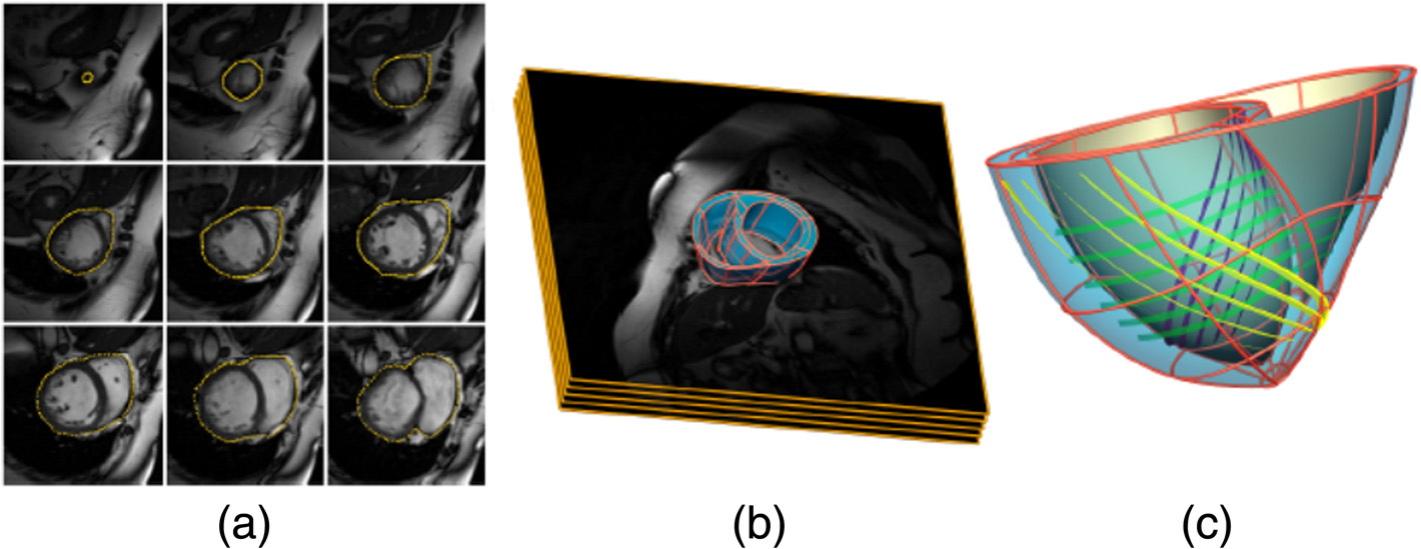
Patient-specific bi-ventricular model. **(a)** *In-vivo* cardiac MRI slices showing manually segmented epicardial contour. **(b)** 3D cardiac model overlaid on the MRI stack. **(c)** Finite-element mesh with tri-cubic Hermite elements showing the main direction of fibre orientation at epicardium (*yellow*), midwall (*green*) and endocardium (*purple*). Reproduced with permission from [39]

The increasing availability of *in-vivo* cardiac images together with the rising trend towards personalised medicine resulted in the definition of *patient-specific models*. They model the cardiac anatomy of a specific human subject from *in-vivo* images, usually MRI [37-39] or CT [40,41]. Figure 1 shows a *patient-specific* bi-ventricular model built from *in-vivo* MRI [39]. Building this kind of model requires imaging techniques synchronised with the ECG and breathing in order to overcome the noise and motion artefacts due to the cardiac cycle and breathing movements. This has also enabled building *dynamic models* that include the intra-subject anatomical variations of the heart due to the cardiac cycle [37,38].

*Cardiac atlases* also emerged thanks to the increasing availability of *in-vivo* images. They are assembled by averaging several 3D cardiac image datasets from a population of subjects, thus generating a mean 3D cardiac image or shape (for further details about cardiac atlases see *Cardiac image segmentation* section). For instance, the cardiac atlas developed in [42] was constructed from 14 manually segmented cine-MRI images and in [43] *in-vivo* multislice-CTs (MS-CT) from 100 subjects were used.

**Figure 2:**
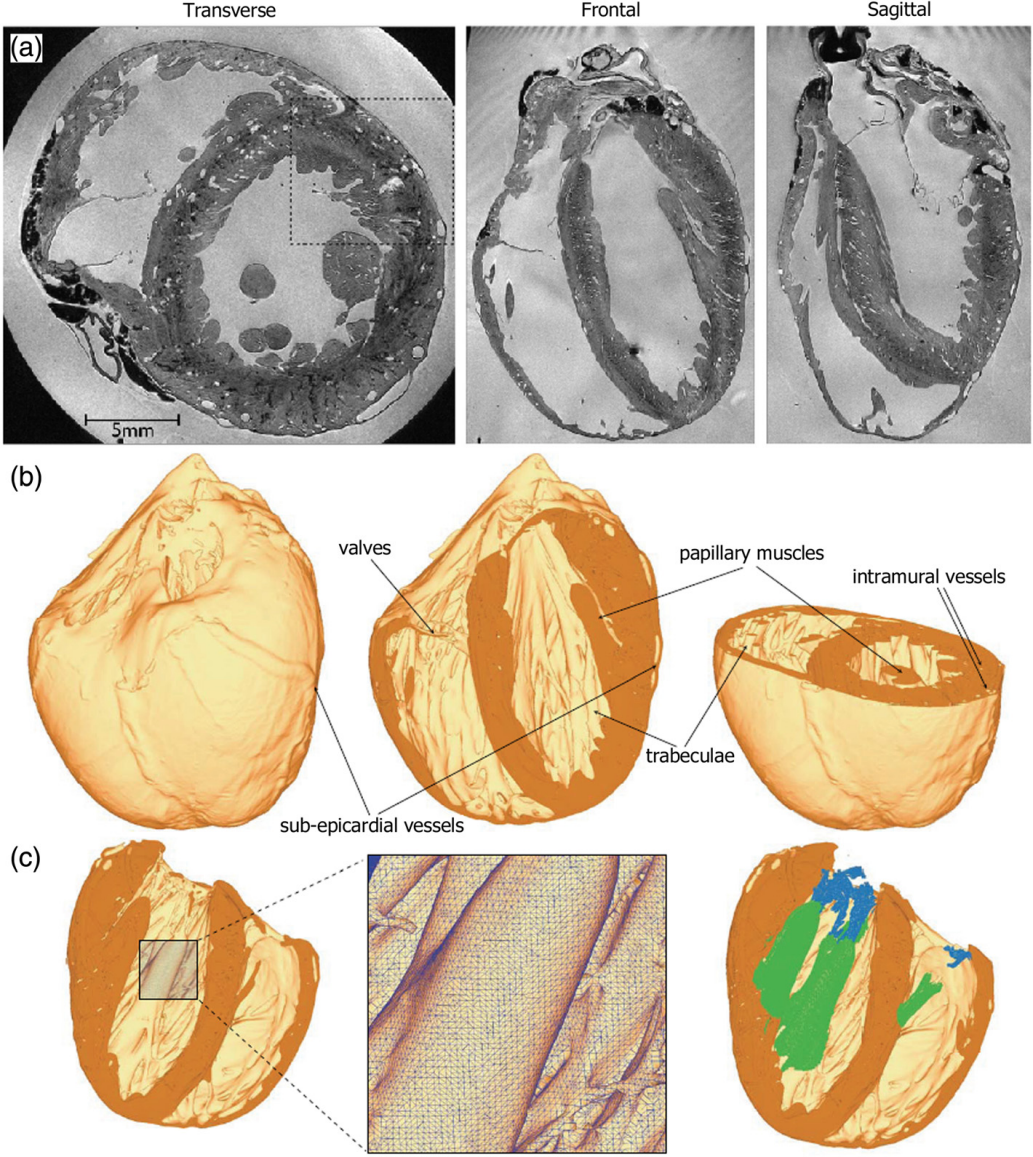
Highly-detailed rabbit bi-ventricular model. **(a)** Very high resolution *ex-vivo* MRI. **(b)** 3D rendering of the model showing a high level of anatomical detail. **(c)** Detail of tetrahedral finite-element mesh showing the papillary muscles *(green)* and chordae tendineae *(blue)*. Adapted with permission from [46].

There are a few *highly-detailed bi-ventricular models* built from very high resolution *ex-vivo* MRI datasets (~25 μm per slice) from small mammalian hearts, which show an outstanding level of anatomical detail including papillary muscles and endocardial trabeculations. Some of them even take into account detailed information at tissue level provided by histological slices with specific staining [44,45]. Figure 2 shows an example of a highly-detailed rabbit bi-ventricular model [46].

## Elements of a 3D cardiac computational model

**Figure 3:**
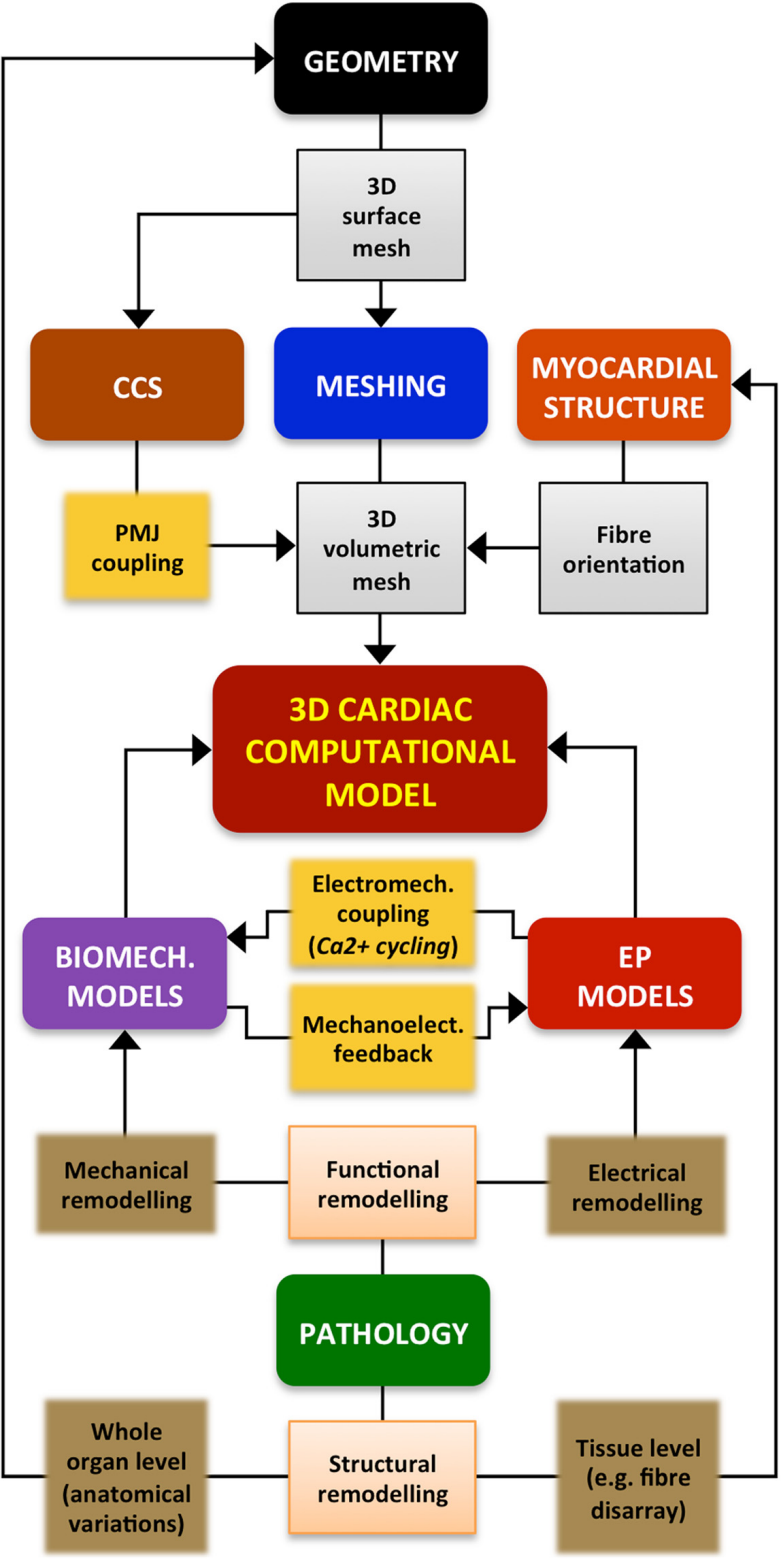
Full pipeline to build a 3D cardiac computational model aimed at biophysical simulation. Summarised flowchart showing the main stages of the construction of a 3D cardiac model aimed at biophysical simulation: 3D cardiac geometry generation, meshing, CCS generation, myocardial structure generation, biophysical modelling (cardiac EP and biomechanics) and cardiac pathology modelling. Lines and arrows depict the relationships between the different stages by means of partial results (*grey boxes*) and coupling steps (*yellow boxes*). For pathology modelling, the diagram shows the different types (*orange boxes*) and subtypes (*brown boxes*) of cardiac pathology that can be included in a cardiac computational model and the stage in which each type of pathology must be taken into account.

In addition to the 3D geometry representing part of the cardiac anatomy, every 3D cardiac computational model may also require other elements, such as the structure of the cardiac tissue, biophysical models of the heart (EP and/or mechanical), pathologies that affect the myocardium, etc. In this section we review the data sources and computational methods used to include those elements into a model, also specifying which of them are necessary depending on the final purpose of the model. Figure 3 shows a flowchart depicting the full development pipeline of a 3D cardiac computational model aimed at biophysical simulation, showing the main stages of the building process and the relationships between them. These steps will be addressed in the following sections, mainly focusing on cardiac EP simulation and providing an extended diagram specific to each step.

### Geometry

As shown in Figure 3, the generation of a 3D cardiac geometry, usually represented by a 3D surface mesh, is the very first step of the construction process of a 3D cardiac model. The geometry of the heart is a key feature that must be represented by 3D cardiac models accurately and realistically. In general, the geometry of a 3D model represents one or several cardiac chambers (LV, bi-ventricular, atrial or whole-heart models) and can also include other details such as the great cardiac vessels including outflow and/or inflow tracts [17,47,48], the fibrous annulus of atrioventricular valves [49,50], part of the coronary tree, or some endocardial details such as papillary muscles and trabeculae carneae for ventricles or *crista terminalis*, pectinate muscles and *fossa ovalis* for atria [21,24,25]. However, it is important to note that the completeness and the anatomical realism and accuracy required by a particular 3D cardiac model will strongly depend on its final application. In [46] it was concluded that structurally simplified models (without endocardial details or vessels) are well suited for a large range of 3D cardiac modelling applications aimed at EP simulation, although the presence of trabeculae provides shortcut paths for excitation causing regional differences in electrical activation patterns after pacing compared to anatomically non-detailed models.

**Figure 4:**
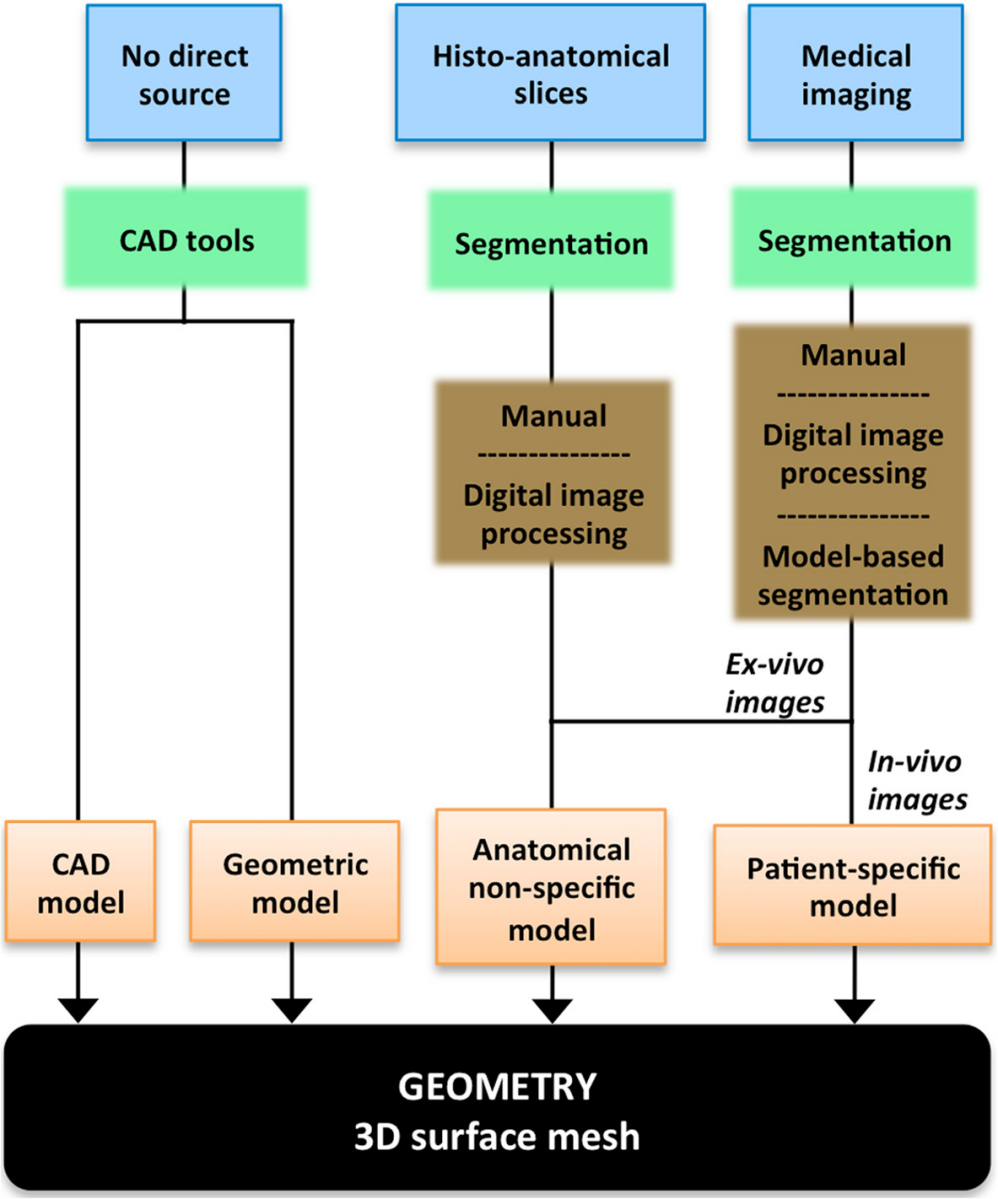
3D cardiac geometry generation stage of the development process of a 3D cardiac computational model. Diagram depicting the main alternatives to generate the 3D surface mesh that represents the cardiac geometry, showing the sources of anatomical information (*blue boxes*) and the methods (*green boxes*) with their possible options (*brown boxes*) used for this task, as well as the kind of model (*orange boxes*) obtained by each method.

The level of anatomical detail achieved by a given model also depends strongly on the source of anatomical information and the methodology used to build it, as shown in Figure 4. Geometric or CAD models, whose geometry shows a coarse representation of cardiac anatomy, are built from population-based data just taking into account some measurements of cardiac chambers volume or wall thickness [4,18]. They are normally used when no direct source of anatomical information is available or when the simplicity of the geometry is preferred to the anatomical realism for the purpose of the model [6,7,22]. Histo-anatomical slices can provide highly detailed anatomical [25] and also histological information [44,45]. However, there is usually a large gap between adjacent slices what leads to the loss of great amount of information out of plane [9,10,14], although it can be mitigated by means of interpolation techniques.

Medical image-based models can include *patient-specific* details obtained from clinical imaging data and/or population-based properties collected from *ex-vivo* datasets (see Figure 4). Clinical imaging protocols usually provide sparse datasets with large gaps between slices, as in the case of most MRI modalities (e.g. [38,49,51]), what often leads to the use of interpolation schemes. Nonetheless, due to the advance of the imaging techniques this approach can require the segmentation of large stacks of tomographic images, especially for high-resolution *ex-vivo* datasets (e.g. [32,35,36,46]) or cardiac atlases whose construction involves segmenting numerous *in-vivo* datasets (e.g. [43,52]). Manual segmentation requires expertise and is very time consuming, while automatic segmentation of cardiac images is still challenging, especially for *in-vivo* datasets. Despite this, clinical imaging techniques (mainly MRI and CT) are today the source of anatomical information most commonly used to generate the geometry of 3D cardiac models.

*Ex-vivo* cardiac images can provide much higher spatial resolution than *in-vivo* datasets for several reasons: absence of motion artefacts, removal of surrounding tissue before the scan and lack of the limitations imposed by alive subjects (either human or non-human) regarding the acquisition time and the ionizing radiation dose (in the case of CT modalities). It allows detailed reconstructions of cardiac geometry, including structures very difficult to observe in *in-vivo* images such as Bachmann’s bundle or pectinate muscles in the atria and endocardial trabeculations in the ventricles [31,45] or leaflets of the cardiac valves and the chordae tendineae [46]. Recently, *ex-vivo* micro-CT with iodine staining has allowed reconstructing structures such as the atrioventricular node and atrial preferential conducting bundles [36]. Among the reviewed works, the segmentation of *ex-vivo* images was usually performed by bi-dimensional (2D) semi-automatic approaches (slice by slice) by combining classical image processing methods such as *region growing* [31,35], *snakes* [28,30] or *level sets* [32,34]. However, manual correction was needed in most cases after the automatic segmentation process [30,31,34,35]. For those models based on very high resolution *ex-vivo* MRI, 2D semi-automatic segmentation was also applied but with a lower level of manual interaction, e.g. using *thresholding* and *morphological operators* [44] or complex pipelines based on *level sets* [45,46,53].

*In-vivo* images can provide both anatomical and temporal *patient-specific* information, thus enabling the characterisation of cardiac motion [52,54]. The reviewed *patient-specific models* based on *in-vivo* MRI were mostly assembled by manual segmentation [37,39]. Images provided by certain MRI modalities, such as cine-MRI, can be segmented by 2D automatic approaches combining *morphological operators* and *snakes* [38]. 2D semi-automatic approaches based on *snakes/level sets* [40] and even 3D automatic methods [41] were applied to *in-vivo* MS-CT. Some *cardiac atlases* were also assembled from manually segmented MRI [51,55]. Nevertheless, to facilitate the segmentation of large amount of datasets, more complex approaches have been applied to assemble *cardiac atlases*: fitting of a deformable model based on geometrical shapes followed by manual correction [56], adaption of an initial mesh by piecewise affine transformation [47] or non-rigid registration with a previously manually segmented image [43,52,54].

In conclusion, high-resolution *ex-vivo* datasets enable much more detailed reconstructions of cardiac anatomy than *in-vivo* ones. However, in addition to the explantation of the heart, the organ must undergo a whole process of tissue preparation (fixation, chambers filling, etc.) before the acquisition of *ex-vivo* cardiac datasets, either *ex-vivo* images or histological slices. This process could alter several features of cardiac structures, such as shape, size, volume, etc., especially in the case of histological sections due to the deformation caused by the slicing process [57-59]. Therefore, even though it is undoubtedly a good approximation, today it still remains unclear to what extent an *ex-vivo* derived geometry is relevant to the *in-vivo* function of the heart, as posed in [26]. To our knowledge, there is no literature addressing this issue thoroughly, so it is something to take into account when a 3D cardiac model is used to carry out computational simulation studies with potential clinical relevance.

Cardiac models can also include the coronary tree, which is often virtually generated from the anatomical knowledge, manually segmented from pictures of histo-anatomical slices [17] or fitted from a previous model [56]. The full coronary tree can be segmented from very high resolution *ex-vivo* MRI [44,46,53]. Using complex segmentation pipelines the main coronary arteries can be reconstructed from *in-vivo* MRI [37]. However, high-resolution MS-CT has become the modality for *in-vivo* assessment of the structure of the coronary tree since it allows segmenting part of the *patient-specific* cardiac vascular network [43,52]. There are some applications in which the coronary tree might have a central role in the model, such as cardiac resynchronisation therapy (CRT) where the implanted leads are spatially restricted to the lumen of some specific veins [60,61]. Other authors have also studied the role played by blood vessels (e.g. fibre orientation changes around vessels) within the heart in stabilising arrhythmias, reporting changes in wavefront curvature around the blood vessels [62].

### Meshing

**Figure 5:**
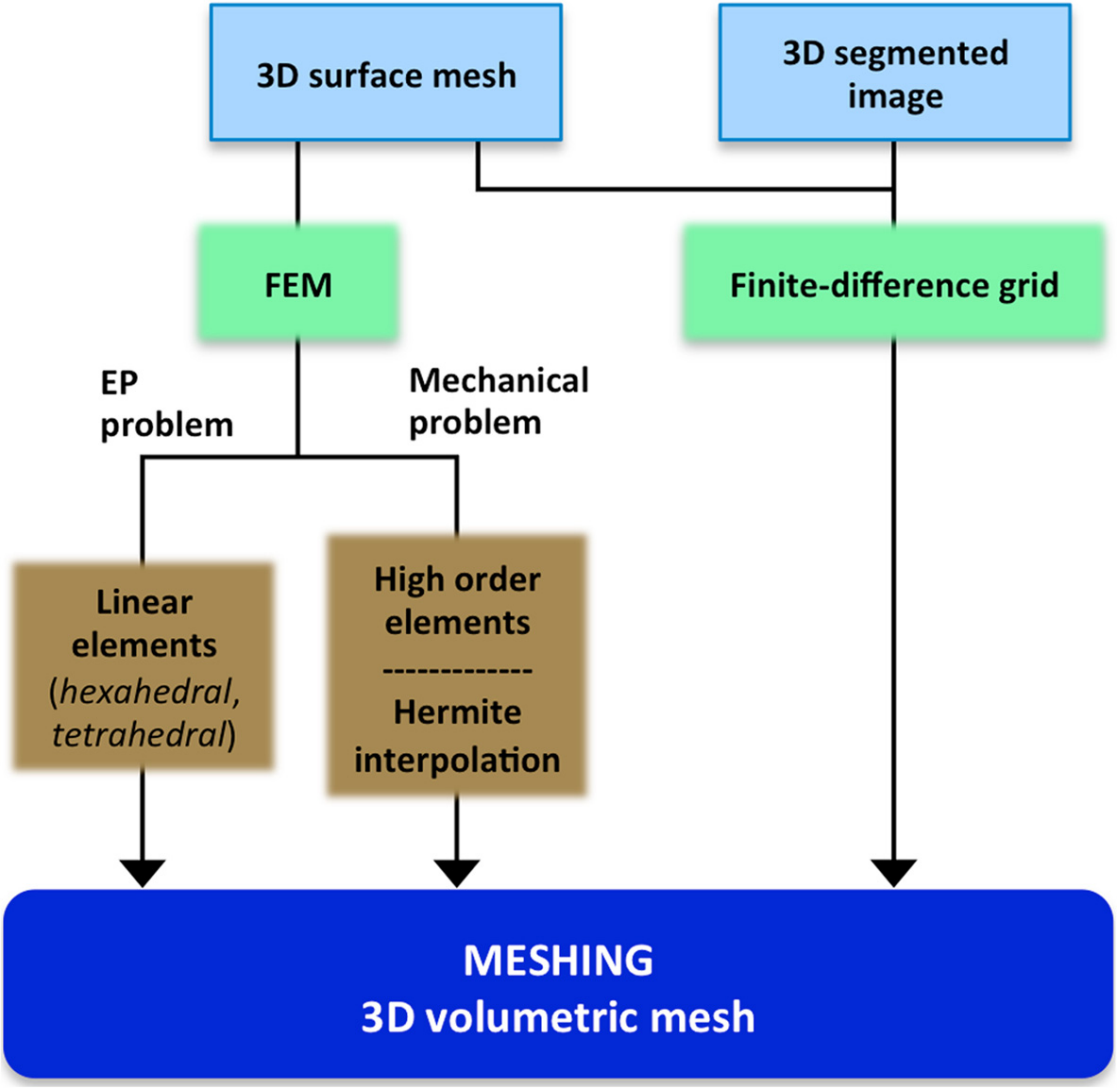
Meshing stage of the development process of a 3D cardiac computational model. Diagram describing the most common methods (*green boxes*) and options (*brown boxes*) to build the 3D volumetric mesh of a cardiac model using the 3D surface mesh or the 3D segmented image resulting from the cardiac geometry generation as a starting point for the meshing process.

Although simple heart models still play an important role for certain applications that focus on mechanistic enquiry, current trends are moving towards *patient-specific* complex anatomical models. Both simple and detailed anatomical heart models are commonly represented by 3D elements resulting from a meshing process. Figure 5 shows an overview of the most common meshing options for 3D cardiac models. The homogenisation of discrete tissue components and the adoption of advanced spatial discretisation techniques based on the finite-element method (FEM) have enabled the resolution of complex biophysical problems. As shown in Figure 3 and Figure 5, anatomical models are usually represented by discrete 3D surface meshes resulting from the geometry generation stage, which will serve as an input for a volumetric mesh generator software (e.g. Tetgen, NetGen, Tarantula). The most common alternative to FEM method is based on grid-based meshes, which can operate directly from a segmented image stack to discretise the volume [45] (see Figure 5).

For EP simulations, unstructured volumetric FEM meshes are commonly used consisting of linear elements that are usually tetrahedral [46], hexahedral or a combination of both [63]. The use of hexahedral elements is desirable to decrease the number of degrees of freedom of FEM models, at the cost of a poorer representation of cardiac anatomy [33,64]. Another extended representation of cardiac anatomy uses cubic Hermite elements, which provide a smooth representation of the model geometry that is well-suited to simulate large deformation mechanics [65]. Although that representation fails to faithfully represent the subtle anatomical details present on the heart, it shows a higher numerical accuracy for mechanical simulations than linear interpolation schemes in models based on tetrahedral or hexahedral elements [66]. Indeed, models aimed at electromechanical simulations usually include two coupled FEM volumetric meshes: one based on linear elements to solve the electrical component and one based on higher order elements [6] or Hermite interpolation functions [34] for the mechanical problem.

The equations to be solved on FEM models impose strong restrictions on mesh elements. In addition, the inclusion of fine anatomical structures (Purkinje, trabeculae, vascularisation) to faithfully represent the cardiac anatomy also increases the number of degrees of freedom of a model. Spatial (ds) and temporal discretisation (dt) constraints are imposed when biophysical models are used, which are in the order of ds = 0.1-0.5 mm and dt = 0.05-0.005 ms [67]. The main reason is the fast upstroke of cellular depolarisation, which produces a step-like wavefront over a small spatial extent [68]. For the case of phenomenological models, such as *Eikonal* ones, spatial and temporal discretisation is less demanding (order of ds = 0.5 mm, dt = 1 ms), resulting in faster computation times.

### Myocardial structure

Cardiac myocytes are elongated cells arranged in a laminar sheet organisation to form the ventricular myocardium [69,70]. The direction of the longitudinal axis of cardiac myocytes, known as *fibre orientation*, strongly determines the electrical activation pattern of myocardium since the electrical propagation is 3 to 4 times faster along this axis than in the transversal one [71]. Furthermore, myocardial contraction is characterised by a shrinkage along the longitudinal axis of myocytes, so fibre orientation has also a great influence on the mechanical behaviour of cardiac tissue. Thus, fibre orientation must be included in models aimed at performing realistic EP and/or mechanical computational simulations. Once the 3D volumetric mesh resulting from the meshing stage is built, the fibre orientation may be included in the 3D model by setting the direction of the longitudinal axis as a property of every volume mesh element (see Figure 3).

**Figure 6:**
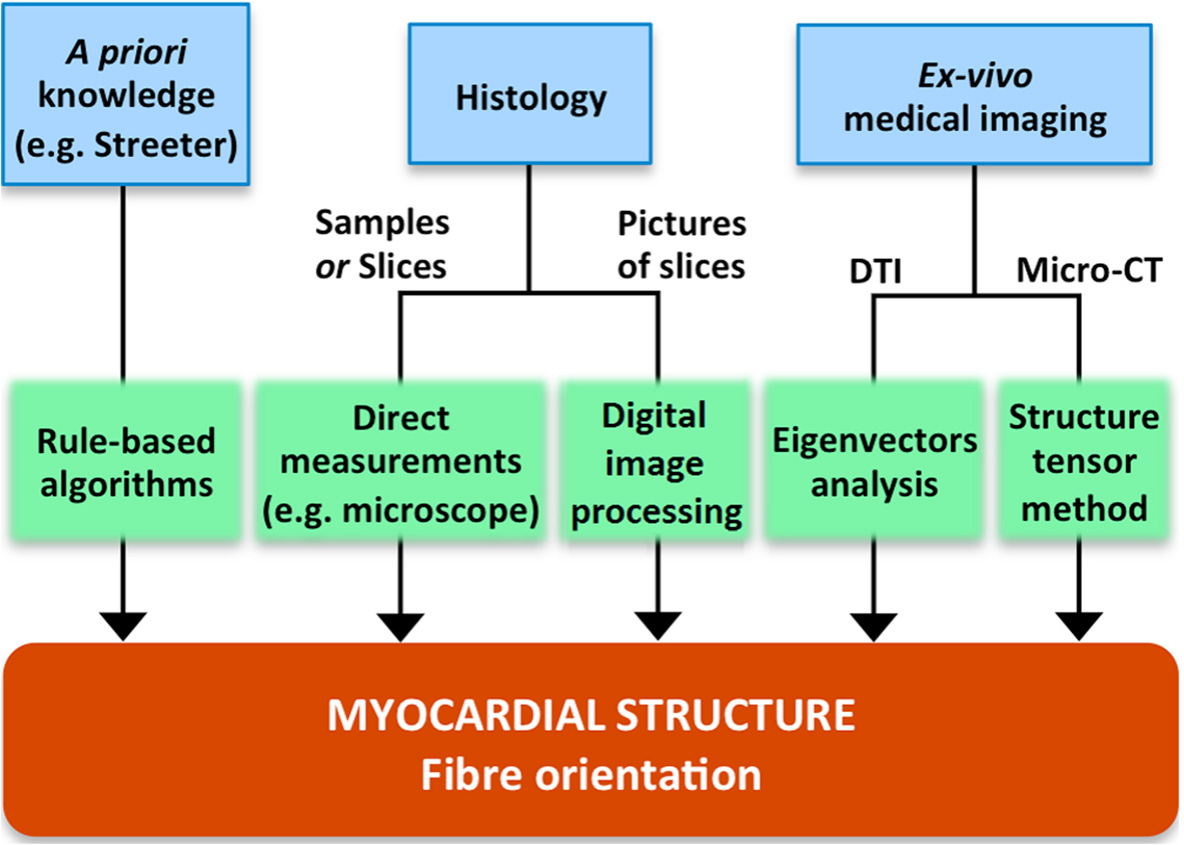
Myocardial structure generation stage of the development process of a 3D cardiac computational model. Diagram showing the main sources of structural information at tissue level (*blue boxes*) and the methods (*green boxes*) used to obtain the fibre orientation to be included in a 3D cardiac model.

Figure 6 shows a schematic summary of the methods most commonly used to obtain the fibre orientation of myocardial tissue. The most usual approach is based on rule-based algorithms that estimate the fibre orientation associated to each element of the volumetric mesh of a model from pre-established patterns [5,6,41,43], most of them derived from Streeter’s findings [72]. Fibre orientation can also be obtained from measurements taken on explanted hearts [12,35], by analysing histological sections under microscope [11] or by digital processing (*structure tensor method*) of volume images assembled from high-resolution pictures of very thin histological slices [25,44].

**Figure 7:**
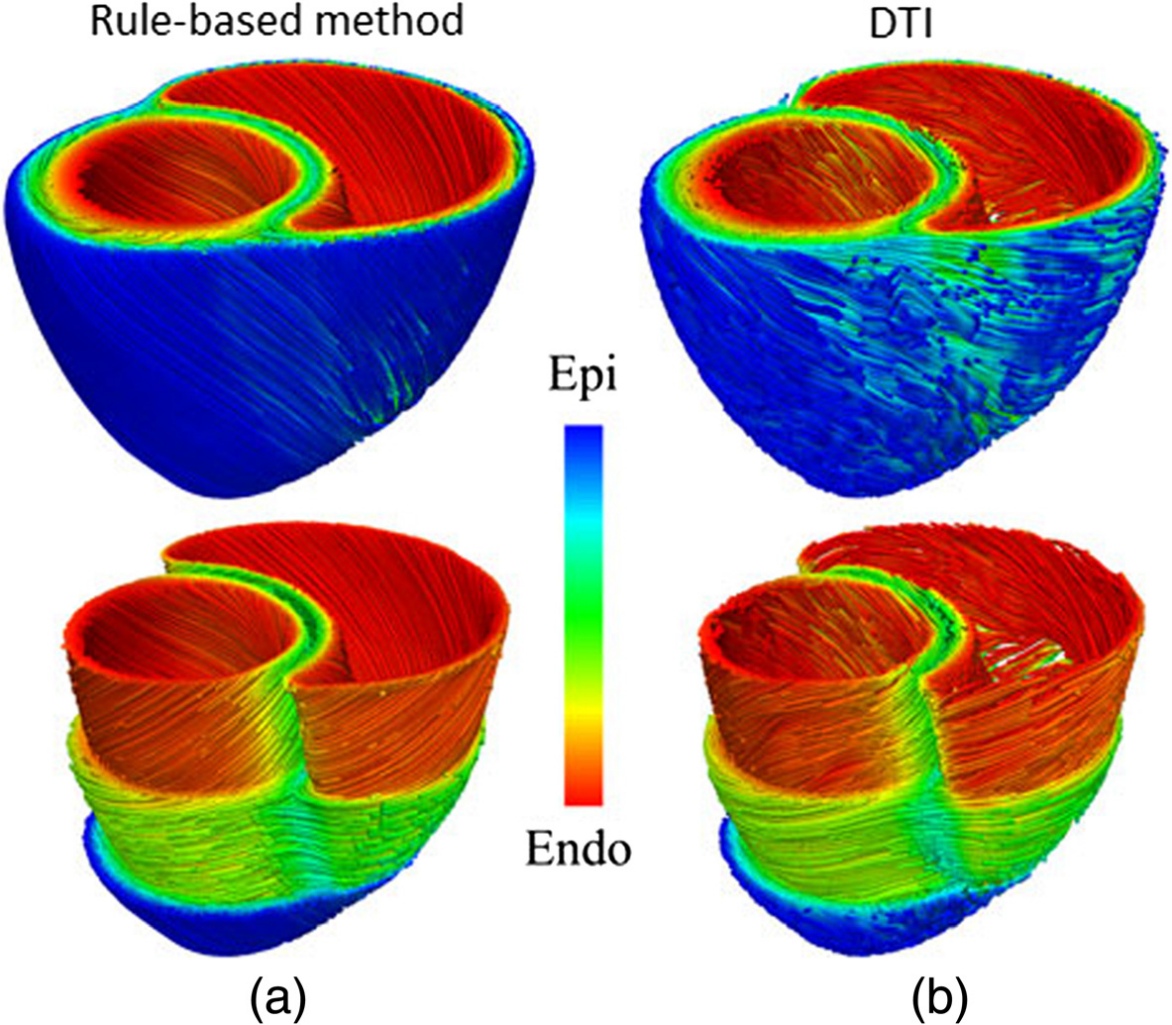
Different methods to include the fibre orientation in 3D bi-ventricular models. Comparison between **(a)** rule-based method (Laplace-Dirichlet) and **(b)** DTI-based estimation of the myocardial fibre orientation for a 3D model of canine ventricles. Adapted with permission from [81].

Diffusion tensor*-*MRI (DT-MRI), also called diffusion tensor imaging (DTI), is a MRI modality capable of showing the diffusion of water molecules within the biological tissues. For cardiac DTI, it is well known that the direction of the primary eigenvector associated to each voxel of the acquired images matches the longitudinal axis of cardiac myocytes [73-75]. This information can be mapped onto the volumetric mesh of a 3D cardiac model to include fibre orientation [31,34,45,76]. In [77] a statistical atlas that characterises the variability of fibre orientation was constructed using *ex-vivo* DTI from nine canine hearts. In recent works there have been proposed approaches to estimate the *patient-specific* fibre orientation of the LV from sparse *in-vivo* 2D DTI slices [78,79] benefiting from the aforementioned fibre statistical atlas [77]. *Ex-vivo* cardiac DTI can also provide anatomical information, thus avoiding the need to merge different image modalities to construct a 3D cardiac model including fibre orientation [30,33]. However, due to its high sensitivity to motion artefacts, *in-vivo* cardiac DTI is not capable of providing the full *patient-specific* fibre orientation of the whole heart yet. In [80] it was shown that global electrical activation patterns obtained by computational simulation from a model with fibre orientation based on a rule-based linear approach were very similar to those based on DTI for the same geometry, thus demonstrating the robustness of the former method for EP simulation studies. Likewise, in [81] a novel rule-based algorithm was compared to DTI-derived fibre orientation (see Figure 7) reaching similar conclusions. Micro-CT with iodine staining is another image modality recently used to assess the fibre orientation in certain critical regions of atrial tissue by *structure tensor method* [36]. Nevertheless, *in-vivo* micro-CT is not feasible because of the needed high dose of ionizing radiation. In conclusion, currently there is no *in-vivo* technique capable of providing the full *patient-specific* fibre orientation of the whole heart.

### Cardiac conduction system

The electrical activation of the ventricles is triggered by a heterogeneous complex 3D structure network that combines subendocardial and free-running fibres forming the so-called *cardiac conduction system* (CCS). The ventricular portion of the CCS ensures the coordinated activation of the ventricular myocardium to achieve the most efficient pumping activity [82]. The His-Purkinje system is highly specialised for a rapid conduction (~2-4 m/s) of the action potential (AP). The CCS is isolated from surrounding myocardium by connective tissue sheaths along its path with the exception of the terminal portion, allowing AP to propagate to ventricular working myocardium at the Purkinje-myocardium junctions (PMJs) [83]. The location of these PMJs plays a key role since they are the source points of the activation of the ventricles [82]. A number of EP computational studies have already integrated CCS models to analyse their role in non-physiological scenarios such as during ventricular tachy-arrhythmias [84,85], ventricular pacing [41] or administration of antiarrhythmic drugs [64]. All these studies reproduce Purkinje cell EP using tailored AP models [86,87] that capture their main electrical behaviour. The most recently developed Purkinje EP models include the Ca2+ subsystem spatial organisation and receptors distribution that are unique to Purkinje cells [88], allowing a more accurate modelling of arrhythmias.

**Figure 8:**
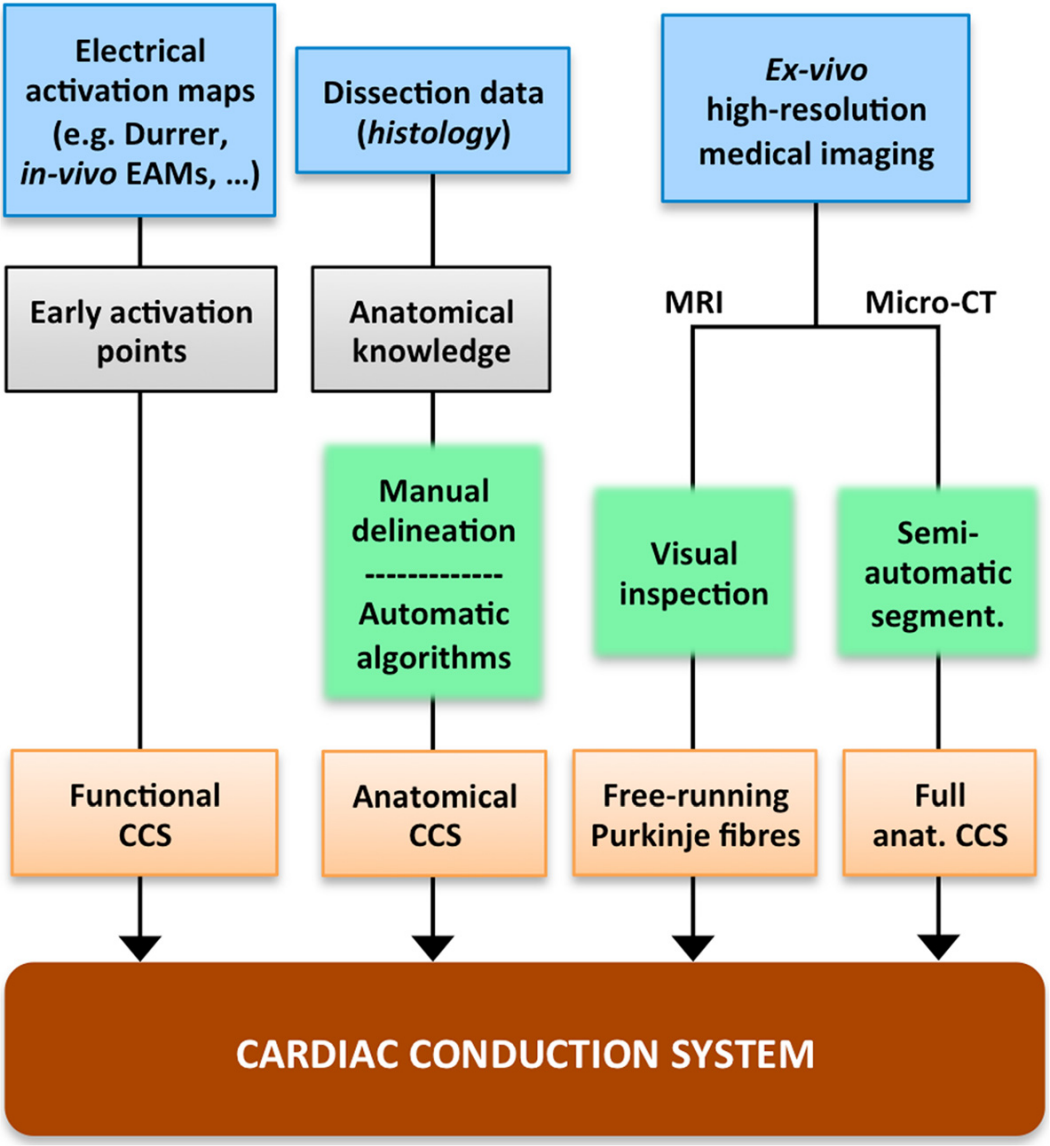
CCS generation stage of the development process of a 3D cardiac computational model. Diagram outlining several ways to generate a model of the CCS to be coupled to a 3D cardiac model. It shows the information sources (*blues boxes*), the partial results obtained (*grey boxes*) and the methods (*green boxes*) used to generate different kinds of model of CCS (*orange boxes*).

Figure 8 shows the main steps and alternatives to generate a model of the CCS that can be coupled to a 3D cardiac model, as shown in Figure 3. The CCS can be visualised using *ex-vivo* microscopic images with specific markers such as stains or connexin antibodies, but cannot be reconstructed from *in-vivo* imaging techniques since its structures are under the level of resolution of current clinical imaging systems. The methods to include the CCS function in computational models are often based on altered endocardial properties that emulate faster activations [89], the setup of a number of early activation points obtained from the literature (e.g. from [90]) or electrical recordings [91], or simplified models that aim to emulate the structure of the CCS network. The latter models can be constructed using different techniques such as manual delineation of CCS on the endocardial surfaces of a 3D cardiac model [41], fractal tree-like representations [92], or algorithms based on the characterisation of the main features of the CCS structure obtained from *ex-vivo* population data by means of histological studies of animal hearts (rat, rabbit, dog or lamb) [93]. Very high resolution *ex-vivo* MRI has allowed locating a number of free-running Purkinje fibres by visual inspection [53,94] and recently *ex-vivo* micro-CT with iodine staining has enabled an image-based semi-automatic reconstruction of the full CCS [95], all in small mammalian hearts.

It is important to highlight that due to the special inhomogeneous coupling between CCS and ventricular working myocardium at PMJs, specific computational models are required for the PMJ coupling (see Figure 3). Those models try to reproduce the propagation delay at PMJs in healthy and pathological conditions, adding transitional regions or resistor elements to couple both cell types [84,96-98]. However, it is still very difficult to determine the location and density of PMJs, and therefore computational studies can provide insights into this important matter in normal and pathological scenarios [85]. Recently, novel methods to estimate the location of PMJs clusters and the structure of the CCS from *in-vivo* electroanatomical maps (EAMs) have appeared [99,100].

### Electrophysiology

**Figure 9:**
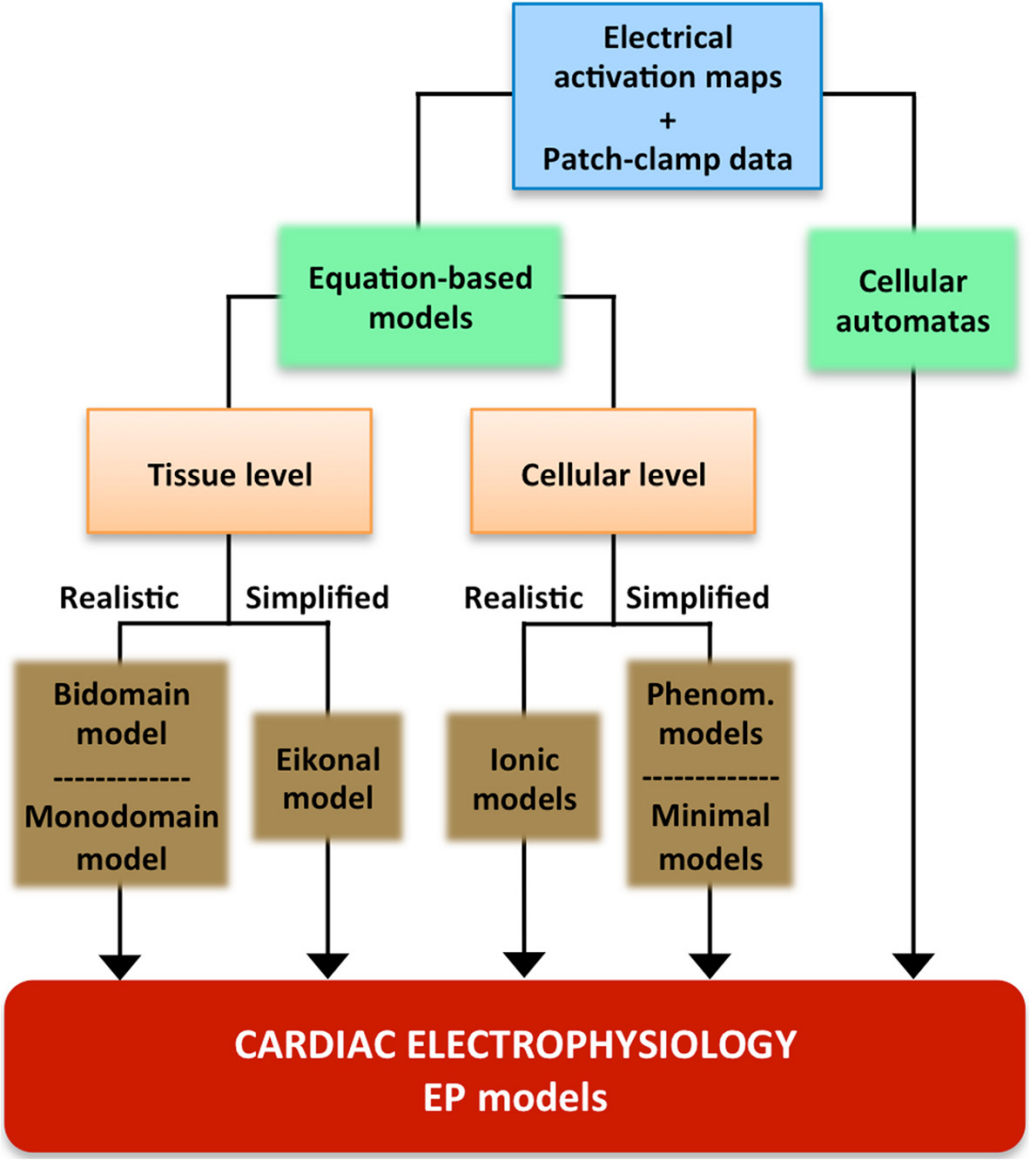
Cardiac electrophysiology modelling stage of the development process of a 3D cardiac computational model. Diagram summarizing very briefly the main methods (*green boxes*) and options (*brown boxes*) to model the cardiac EP by means of EP models from EP data (*blue box*) provided by electrical activation maps at tissue level and patch-clamp experiments at cellular level.

For models aimed at EP computational simulation, once the anatomy and structure of the heart have been defined, a mathematical model that simulates the EP behaviour of the myocardium must be plugged in. Figure 9 briefly summarises the main methods and options to model the cardiac EP using EP models. In the 70s and the 80s, these models usually had the form of *cellular automatas* [10,101], but these rule-based models were progressively substituted by equation-based ones that consist of two parts: the cellular-level equations and the tissue-level equations (see Figure 9). The cellular-level equations are based on the well-known Hodgkin and Huxley (HH) formalism established more than 60 years ago [102]. According to this formalism, the cellular AP and the underlying ionic currents are described by a system of non-linear first order ordinary differential equations (ODEs) that models the kinetics of individual ionic channels, pumps and exchangers and the electrical interaction thereof [103]. While these currents are still formulated using the HH formalism, a new paradigm based on Markov-type models is also being adopted to build more biophysically-based models of ion channels [104]. Over the past decades, extensive patch-clamp experiments that reveal the dynamic properties of ionic channels [105] have provided data to formulate comprehensive mathematical descriptions of ionic currents of different animal species, heart portions and pathophysiological conditions. EP models are now highly specific and include human atrial [106], ventricular [107] and Purkinje cells [87] in normal or diseased conditions (see [103] and [108] for review).

The ventricular wall is not homogeneous, as cardiac myocytes in different portions of the ventricles exhibit different ionic currents and APs. Electrophysiological heterogeneities in the ventricles include epicardial-endocardial [109], apico-basal [110] and left-right [111] differences in ion channels and thus in APs. Recent AP models include some of these regional differences; for instance, the last human AP model by O’Hara *et al.* includes different formulations for endocardial, epicardial and midmyocardial cells [107]. Although there is no experimental evidence on the boundaries of these different regions in the heart anatomy, transmural and other regional differences (e.g. Bachmann’s bundle, crista terminalis or pectinate muscles in the atria) in the EP level have been roughly included in some 3D cardiac models [33,112,113].

Myocardial cells are electrically coupled so that current can flow from one cell to neighbouring ones. Thus, the above mentioned cellular models must be accompanied by a tissue-level model in which axial currents flow between cells through low-resistance *gap junctions*. Such model should take into account the anisotropy inherent to the myocardium, and also the fact that the muscle tissue includes both intracellular and extracellular media (domains) separated by cell membranes. A mathematical homogenisation of this concept leads to the so-called *bidomain model*, which was developed in the late 70s [10]. This model consists of two partial differential equations (PDEs) from which extracellular and intracellular potentials can be derived. Because membrane potentials depend on ionic currents, the tissue-level equations are coupled to the cellular-level ones, so the complete model is formed by a system of two PDEs and a certain number of ODEs, which are strongly non-linear.

The two PDEs include the intracellular and the extracellular conductivity tensors determined by fibre orientation. If equal anisotropy ratios are assumed for the intracellular and extracellular domains (i.e. the two tensors are related by a constant), then the bidomain formulation is reduced to the so-called *monodomain* approach and the two PDEs become uncoupled [114]. One of the PDEs is of the reaction-diffusion type and includes all the ionic current ODEs in its independent term. Membrane potentials can be obtained by solving this system, while extracellular potentials can be derived directly from the second ODE once membrane potentials are known.

The number of state variables in the ionic AP models (and thus the number of ODEs) can be as high as 48 [107]. The systems of PDEs plus ODEs pose a mathematically challenging problem which must be solved using numerical methods, e.g. FEM method, and which is highly computationally demanding. As a result of these demands, high performance computing techniques are ordinarily used to implement these models. To overcome this limitation, simpler alternatives have been proposed at the cost of losing realism in the mathematical description, such as using the so-called *phenomenological models*, which reduce the number of state-variables substituting the actual ionic current descriptions by simple mathematical equations. Historically, the first of these simplified models was developed in 1961 by FitzHugh [115], and this approach was further improved and adapted to cardiac AP [116,117]. The mathematical terms of these models have no biophysical correlates in the form of ionic currents, and thus are not feasible to simulate complex dynamical patterns of excitation and repolarisation of cardiac tissue (e.g. rapid pacing and reentrant activity). The so-called *minimal models*, an evolution of the purely phenomenological models, try to partially overcome this limitation by associating each term to actual but simplified ionic currents [118,119]. A different simplification that affects the propagation part of the phenomenon can be adopted by using the so-called *Eikonal* approximation, which replaces the reaction-diffusion equation with an eikonal equation that is simpler and based on a Huygens approach [120,121]. Recently, a new strategy that combines both assumptions (minimal plus *Eikonal* models) has been proposed [122]. This approach gives rise to a hybrid framework which may combine models with different levels of detail (including detailed biophysical models) whilst maintaining relatively low computational demands [123].

### Electromechanical coupling

Although mentioned, modelling of cardiac mechanics is not addressed in a specific section of this work. Very briefly, it involves the use of biomechanical models at organ level, using the equations of continuum mechanics to describe the deformation of the organ produced by the active tension generated by the myocytes, and models of myocyte contraction at cellular level that include myofilaments models at subcellular level depicting the actin-myosin interactions and its calcium-based activation system (e.g. [124,125]) (see [126] for a review).

However, it is important to highlight that every 3D cardiac model aimed at electromechanical (EM) computational simulation must include the EM coupling, also known as excitation-contraction coupling [127,128], as shown in Figure 3. The electrical activation of myocytes is the event that triggers their mechanical contraction by means of the Ca2+ cycling (the release and reuptake process of intracellular Ca2+), which is the responsible for the initiation of actin-myosin interactions that ultimately lead to myocyte shortening [129]. On the other hand, EM simulation-oriented models can also include the mechanoelectric feedback (see Figure 3). Acute changes in ventricular mechanics can affect cardiac EP [129,130] due to mechanisms such as stretch-activated ion channels [131,132] or mechanical modulation of cell calcium handling, even causing mechanically triggered arrhythmias [133].

### Pathology

There are many diseases that cause structural and/or functional cardiac remodelling, which affects cardiac electrical and/or mechanical performance. Thus, models aimed at studying the effects of those diseases by means of computational simulation should include both types of remodelling. As shown in Figure 3, functional remodelling (electrical or mechanical) may be included in computational simulation-oriented models by adapting the biophysical models that reproduce the behaviour at cell or tissue level. This generally implies altering certain parameters in the equations of the ionic currents to account for the modifications exerted by the remodelling of cardiac tissue [134], or the altered values of certain parameters obtained by patch-clamp experiments at cellular level. A similar approach is used in the case of genetic mutations: their effects on cardiac EP can be mimicked by changing the maximum conductance or the kinetic parameters of the ionic currents directly affected by the mutation [135]. Functional remodelling can also affect the EM coupling. In heart failure, for instance, Ca2+ cycling is altered resulting in impaired contractility [129,136], what increases the risk of extrasystoles and arrhythmia due to the mechanoelectric feedback [137,138]. These arrhythmogenic mechanisms may be studied by 3D computational simulations [139].

Structural remodelling can affect cardiac anatomy at organ or tissue level. A pathological variation of cardiac anatomy, which could affect the volume, shape and/or wall thickness of any cardiac chamber, can be taken into account by the geometry of a model, as shown in Figure 3. There are examples of models showing left atrium dilation due to sustained atrial fibrillation [21], right ventricle (RV) hypertrophy [33], hypertrophic and dilated cardiomyopathy in LV [41] or LV wall thinning because of ischaemic injuries [50]. On the other hand, some cardiac pathologies can also alter the myocardial structure and function at tissue level (see Figure 3), which affects the electrical propagation patterns and the mechanical performance. For instance, a 3D cardiac model can include left bundle branch block (LBBB) as a functional alteration at tissue level [140]. Myocardial structure remodelling at tissue level, such as fibre orientation disarray associated to ischaemic regions [141], may be also included into a model from *ex-vivo* DTI [31,53] or histology data [142].

Chronic or healed ischaemic injuries resulting from myocardial infarctions (infarct scars) can be included in order to assess its influence on cardiac performance. These fibrotic lesions are usually segmented from *ex-vivo* DTI by means of fractional anisotropy [31,53], from anatomical MRI based on wall thinning [50] or more frequently from *in-vivo* delayed enhancement*-*MRI (DE-MRI) [143]. Image-based segmentation provides information about the extension and location of an ischaemic injury within the geometry of a given 3D model and also allows differentiating between the core (fibrotic scar) and the border zone (BZ), i.e., the remodelled but still working tissue. However, the associated functional remodelling must be taken into account by the mathematical models used to perform computational simulations, e.g. ionic models for electrical remodelling in the case of EP studies.

Cardiac tissue can also undergo diffuse myocardial fibrosis, which is related to a broad variety of disorders: hypertension, diabetes, dilated and hypertrophic cardiomyopathy, chronic renal insufficiency, atrial fibrillation, etc. The progress of diffuse fibrosis can lead to systolic and diastolic dysfunction [144] and turns the myocardium into a more arrhythmogenic substrate [145]. Therefore, models can include this kind of fibrosis in order to study its influence on the cardiac performance by computational simulation.

### Example of a 3D cardiac computational model

**Figure 10:**
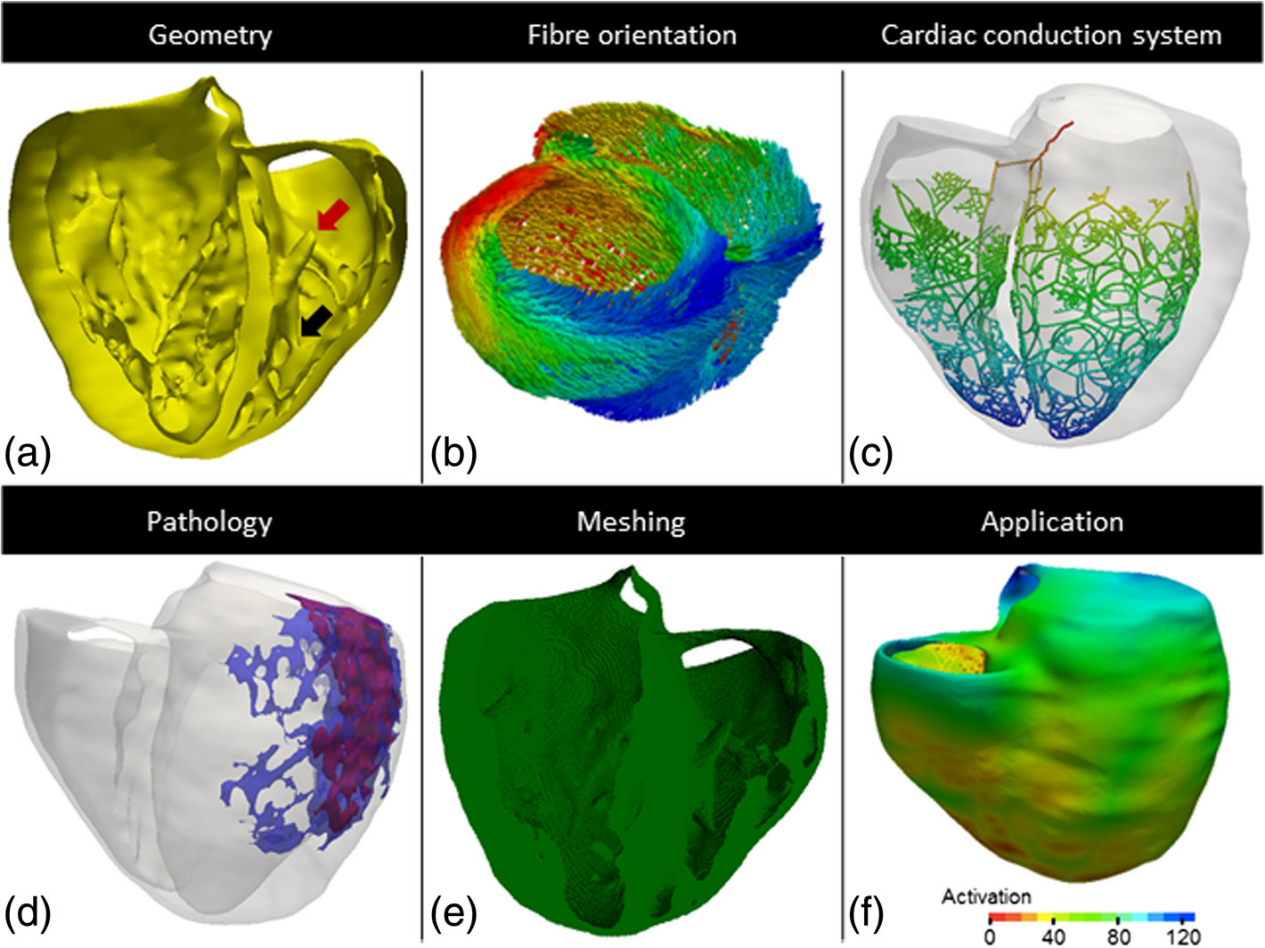
Elements and final application of a 3D bi-ventricular *patient-specific* computational model. **(a)** Geometry of human ventricles with highly detailed endocardium showing details such as the septal papillary muscle (*red arrow*) and the moderator band (*black arrow*) in the RV. **(b)** Fibre orientation from a rule-based algorithm. **(c)** Anatomical model of the CCS. **(d)** Infarct scar showing the core (*red*) and the BZ (*blue*). **(e)** FEM volumetric mesh with regular hexahedral elements. **(f)** Biophysical computational simulation of cardiac EP.

Figure 10 shows an example of a 3D bi-ventricular computational model that includes all the components previously described in this section. The geometry of the ventricles and the scar region are *patient-specific*, both segmented from the same *in-vivo* DE-MRI stack. The cardiac geometry was manually segmented with a high level of anatomical detail (see Figure 10a), whilst the infarct scar was semi-automatically delineated by the standard deviation (SD) from remote method [146]. On the contrary, the CCS, the fibre orientation and the biophysical models are generic, i.e., based on population data. The fibre orientation was included by a rule-based algorithm [147] based on the Streeter’s findings [72]. An anatomical model of the CCS was generated and coupled to the endocardial surfaces of the bi-ventricular model using an automatic algorithm implemented from dissection data [93]. The FEM volumetric mesh was built using regular hexahedral elements (element size (ds) of 0.4 mm) in order to decrease the number of degrees of freedom of the model, yielding a mesh composed by 3.2 million elements and 3.5 million vertices. The final application of this model was computational simulation of cardiac EP using a specific FEM solver called ELVIRA [148], with a time step (dt) of 20 μs, and different ionic models for myocardium (including transmural heterogeneity) [68] and CCS [87] at cellular level, and monodomian approach [114] at tissue level.

## Personalisation of 3D cardiac computational models

*Patient-specific* models can open up a new avenue of possibilities in cardiology since they are able to integrate anatomical and functional information of a given patient provided by a variety of techniques (different imaging modalities, invasive electrical recordings, etc.) in a very comprehensive fashion. This could be very helpful in therapy planning, guidance and follow-up. However, currently only a few cardiac features can be completely personalised. Table 1 summarises the current personalisation possibilities in cardiac computational modelling, which are discussed below.

**Table 1:** **Current personalisation possibilities in 3D cardiac computational modelling**

**Features**		**Technique**		**Invasiveness**
***Anatomy***		Segmentation of *in-vivo* image		Non-invasive
***Fibre orientation***		Image-based estimation (*in-vivo* DTI)		Non-invasive
***Cardiac conduction system***		PMJs from EAMs		Invasive
***Pathology*** (Structural remodelling)	***Anatomical variations***	Clinical image-based		Non-invasive
	***Localised fibrosis***	Image-based (e.g. DE-MRI)		Non-invasive
		EAMs		Invasive
	***Diffuse fibrosis***	Image-based (T1 mapping MRI)		Non-invasive
***Electrophysiology***	***Action potential***	NO		
	***Heterogeneity***	NO		
	***Electrical remodelling***	NO		
	***Genetic mutations***	NO		
	***Conduction velocities***	Global:	ECG	Non-invasive
		Local:	EAMs	Invasive
	***APD restitution curve***	EAMs		Invasive
	***Extracellular ion concentrations***	Blood test (electrolyte concentrations) (*time-variant*)		Invasive
	***Activation pattern***	ECG or BSPM		Non-invasive
		EAMs		Invasive
***Cardiac mechanics***	***Biomechanical model***	Dynamic image-based		Non-invasive
	***Material properties***	NO		
	***Boundary conditions***	Dynamic image-based		Non-invasive

This table shows the techniques that can be currently used to personalise the different features and components of a 3D cardiac computational model aimed at biophysical simulation, specifying whether the technique is invasive or non-invasive.

The anatomy, including pathological anatomical variations, can be personalised for a specific subject by developing a *patient-specific* model from *in-vivo* images. Some types of structural remodelling at tissue level can also be personalised, such as the location and extension of infarct scars which can be reconstructed from *in-vivo* images (e.g. DE-MRI) [143] or from EAMs recorded during RFA procedures. T1 mapping is an emerging MRI modality able to quantitatively assess *in-vivo* the level of diffuse fibrosis [149,150]. This novel technique could allow including the *patient-specific* level of diffuse fibrosis in a 3D cardiac model in a quantitative fashion.

There are two important features that cannot be completely personalised yet: the CCS and the fibre orientation. EAMs can provide the location of some PMJs allowing an inverse estimation of a CCS model that tries to reproduce the *patient-specific* electrical activation pattern [99,100]. Nonetheless, currently there is no *in-vivo* image modality with enough spatial resolution to enable a detailed visualisation of the CCS structure. For fibre orientation, only sparse 2D DTI slices can be acquired *in-vivo* to estimate the *patient-specific* fibre orientation of the LV [78,79].

Regarding cardiac EP, the *patient-specific* transmembrane current dynamics cannot be measured and hence the mathematical models (e.g. ionic models) that reproduce the AP at cellular level cannot be personalised. Due to the same reason, the electrical heterogeneity between different regions (e.g. transmural heterogeneity in ventricular walls), the electrical remodelling or the effects on cardiac EP of a genetic mutation cannot be personalised. However, the EP model at cellular level that best matches the patient’s pathology can be chosen from the existing models, obtaining a *patient-group* instead of a *patient-specific* personalisation [151,152]. The *patient-specific* electrical activation patterns and wave propagation conduction velocities (CV) in ventricular myocardium can be estimated for the *Eikonal* model either globally from ECG or body surface potential maps (BSPM), or locally (as spatially varying parameters) by means of EAMs [153], even taking into account the uncertainty due to the sparsity and noise of clinical data [154]. In [122] an approach to personalise CV and APD restitution curve for bi-ventricular models was proposed by estimating certain EP parameters of a simplified AP model [117] from the patient’s ECG and the isochrones provided by *in-vivo* non-contact endocardial LV mapping [155]. EAMs can also allow locating electrical pathways for a certain subject, such as the main inter-atrial connection [151,152] or the reentrant channels responsible for an infarct-derived ventricular tachycardia (VT) [156,157]. Finally, extracellular ion concentrations can be estimated and set into a model from the measurement of blood electrolyte concentrations, although they are highly time-variant [151,152].

For cardiac mechanics, the most feasible personalisation approach consists of adjusting some parameters of a biomechanical model using the information obtained by segmenting dynamic images (e.g. cine-MRI, tagged-MRI, dynamic-CT), so that the model is adapted in order to reproduce the *patient-specific* cardiac motion as accurately as possible [153]. Boundary conditions can be estimated from dynamic images as well.

## Applications of 3D cardiac computational models

Computational clinical cardiology is currently a rising field of research with a large number of potential applications. Cardiac image analysis/segmentation and computational simulation of cardiac physics are two well-established applications of 3D cardiac models. Cardiac EP simulation, for instance, is becoming a powerful tool to gain insight into the electrical cardiac disorders at tissue/organ level. It allows performing *in-silico* experiments by computational simulation in which all variables are under control and that, in many cases, cannot be carried out *in-vivo* due to unacceptable risk for the patient, ethical reasons, inability to control all variables, etc. In this section we expose the usefulness of these applications by presenting several examples from the literature mainly related to cardiac EP simulation and model-based segmentation.

### Cardiac image segmentation

One of the most challenging tasks in the development of *patient-specific* models is the segmentation of *in-vivo* cardiac images. A wide variety of methods have been developed (see e.g. [158]) but the most advanced approach for the automation of this task is the *model-based segmentation*. This paradigm has been widely applied to *in-vivo* cardiac image segmentation and analysis [159]. It requires the use of a reference model so that several kinds of model especially aimed at this purpose have appeared.

*Deformable models* are based on a template that resembles the target objects, i.e. the cardiac structures to be segmented, which is used as an initialisation of the segmentation process. This template, called *initial mesh*, can be built using any methodology: ellipsoid-based model [7], image-based model [49,76], assembled from dissection data [160], etc. Briefly, the *initial mesh* is overlapped to the cardiac image stack to be segmented and then it is deformed driven by the image intensity levels in an iterative process until reaching a given optimum point with the ultimate goal of fitting the *initial mesh* to the *patient-specific* geometry.

**Figure 11:**
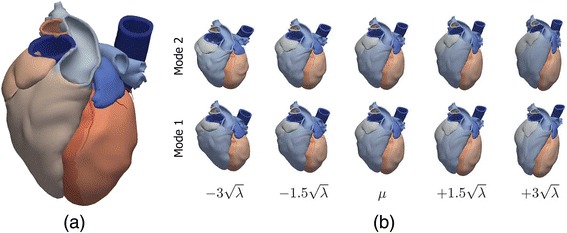
Whole-heart cardiac atlas constructed from *in-vivo* high-resolution MS-CT of 138 human subjects. **(a)** Mean shape of the whole-heart atlas. **(b)** First two modes of variation at end diastole of the *spatio-temporal whole-heart statistical atlas* (*μ* represents the mean shape and $\sqrt{\lambda }$ is the standard deviation). Adapted with permission from [52].

The mean shape/image resulting from a *cardiac atlas* can also be used as a segmentation tool by fitting it to the target image, e.g. by non-rigid registration [42]. The so-called *statistical cardiac models* (or *statistical atlases*) appeared as an evolution of cardiac atlases. They are a wide range of models mainly represented by *statistical shape models* [51,52,54,55], although there are other types such as *active appearance models* [161,162] or *active shape models* [163]. This model-based segmentation approach relies on an *a priori* statistical knowledge about cardiac anatomy and/or certain features of cardiac images. This knowledge comes from a statistical characterisation of the anatomical variations (and/or image features) included in the population used to construct a given atlas, usually including both healthy volunteers (normal shaped hearts) and patients with different variations of cardiac anatomy. Figure 11 shows the mean shape and the statistical characterisation of a whole-heart statistical atlas [52]. The dimensionality of the resulting variance and co-variance matrix is usually limited using principal components analysis (PCA) [47,51,54,55]. The resulting prior knowledge is used in order to enhance the accuracy of the segmentation or analysis process on a specific image modality and to increase its robustness when certain information in the image is poor or missing (see [164] for a review). A mixed approach is the *shape-constrained deformable model* developed in [47], which includes prior anatomical knowledge provided by a point distribution model (PDM) derived from a cardiac atlas. Although the particular method can vary depending on the type of model, in general the segmentation process is guided by the image information but constrained by the prior knowledge. That is why a statistical model does not consider feasible those anatomical variations that were not learned from the training set. Therefore, the power of a statistical atlas as a segmentation tool strongly depends on the population chosen for the atlas development. Furthermore, most of the statistical models take into account the endocardial surfaces of all cardiac chambers included in the model but only the epicardium of the LV [47,51,52]. This is because of the high complexity and variability of the anatomy of the RV and the atria together with the thinness of their walls, what complicates the statistical characterisation of these cardiac chambers. *The Cardiac Atlas Project* [165], led by the University of Auckland, must be highlighted in this section. It provides a wide database of cardiac images available online which aims to promote a collaborative development of anatomical and functional statistical atlases both for healthy and pathological hearts.

### Simulation of acute ischaemia

In the first 10-15 minutes after coronary artery occlusion (acute ischaemia), changes in ionic currents and concentrations, AP and tissue structure, along with the heterogeneous nature of these changes, predispose the ventricular myocardium to potentially lethal reentrant arrhythmias [166]. In the past decades, the intimate mechanisms of the arrhythmogenicity of acute myocardial ischaemia have been theoretically studied using model-based computational simulation [167]. The effects of acute ischaemia are of multiscale nature [168-173], and the whole-organ effects have recently been analysed using 3D cardiac models.

In [174] a 3D heterogeneous model of regionally ischaemic human ventricles was used to study the dynamics of ischaemic reentrant pathways. The simulation results predicted the appearance of figure-of-eight reentrant wavefronts that cross the central ischaemic zone. These wavefronts are formed in the epicardial surface due to the prolonged refractory period of midmyocardial layers. Also, focal activity experimentally observed in the epicardium could be caused by reentrant wavefronts propagating in the mid-myocardium which re-emerge in the heart surface. Finally, the thin survival layer (wash-out zone) which appears in the endocardial ischaemic BZ protects the myocardium against the perpetuation of reentrant wavefronts that cross the central ischaemic zone.

Global ischaemia has been also simulated in 3D cardiac models in the context of cardiac defibrillation. In [175] a 3D model of rabbit ventricles was used to study the effects of ischaemia on the lower and upper limits of vulnerability to reentry induced by electric shocks. These studies stressed the importance of the transmural electrical events, the spatial extent of the shock-end excitation wavefronts and the slower recovery from shock-induced positive polarisation in the mechanisms responsible for the limits of vulnerability.

### Ablation of chronic myocardial infarction

When the acute phase of ischaemia ends, the ischaemic (now infarcted) tissue heals and ventricular arrhythmias can take place weeks or months after the occlusion [176]. Computer simulations are also of great interest in this chronic period of infarction to aid clinicians during ablation interventions. Examples of simulations of infarcted 3D ventricles are discussed below.

In [177] reentrant mechanisms were simulated on a 3D image-based model of canine infarcted ventricles, including the scar and the BZ with electrical remodelling. The mechanisms of defibrillation efficacy were studied in [178] using a model of rabbit infarcted ventricles. The ability of computer simulations based on DTI images to predict the VT circuits measured in swine EP studies was shown in [179]. Similarly, electrically remodelled BZs and infarct cores were included in [180] in a model of swine heart. VT was induced in different computational models and proved that both infarct scars and peri-infarct zones are needed for VT generation. Similar reentrant circuits were obtained both in computational and EP studies, showing that image-based modelling might be helpful in planning RFA strategies. Indeed, recently the feasibility of 3D simulation based on *in-vivo* DE-MRI images to estimate ablation targets in human VT has been tested [143,181], emphasizing the effectiveness of this computational tool.

### Cardiac resynchronisation therapy

CRT has become an established therapy to treat certain patients with heart failure suffering from conduction abnormalities such as LBBB. Clinical trials evaluating CRT have demonstrated that 30% of patients with heart failure and wide QRS do not respond to CRT, especially patients with myocardial infarcts. Computational models have been developed to provide insight into the complex mechanism that governs CRT efficacy. In [140] a computer model was designed including different degrees of LBBB (0%, 20% and 40%) for the optimisation of atrioventricular and interventricular (VV) delay, which are key parameters on the CRT device that require a tailored set-up. In [182,183] the effect of the pacing site and infarct location (anterior, inferior, posterolateral, diffuse fibrosis) on regional mechanics and global haemodynamics was studied on an electromechanical dog heart model. They concluded that in hearts with LBBB and large infarcted regions the response to CRT was poorer. The effect of the ventricular morphology (normal, dilated, hypertrophy) on the VV delay in order to obtain an optimal LV synchronisation was studied in [41]. They concluded that the distance between the LV lead of the pacemaker and the CCS (related to thickness of the LV lateral wall) introduces a large delay that needs to be compensated by a pre-activation of the LV lead. In [184] a personalisation strategy for anatomy and function was developed to predict the response to CRT *in-silico*. Personalised heart models reproduced acute effects of pacing on pressure development for several pacing conditions on two patients, achieving good agreement with invasive haemodynamic measurements. All those computer models were designed with the aim of helping to understand the pathophysiology of asynchrony to further improve CRT.

## Conclusions

3D computational models of cardiac anatomy and function have benefited significantly from the revolution of medical imaging systems. The development of techniques able to build 3D personalised cardiac models is expected to have an impact on diagnosis, therapy planning and prevention of cardiac disorders. The advances on *patient-specific* modelling have enabled the use of 3D heart models reconstructed from clinical MRI or CT scans. Current 3D cardiac models have a remarkable structural and biophysical detail, and provide a multi-parametric source of information that integrates multimodal images (*ex-vivo* and *in-vivo*), one-dimensional signals and biophysical data into a common spatio-temporal coordinate system, which will help to gain insights into several cardiac disorders, e.g. into mechanisms of arrhythmia in many disease settings. Incorporation of critical cardiac structures, such as the CCS, fibre orientation and the coronary tree, will facilitate further biophysical modelling. The next steps towards the translation of this technology into clinical environments are the automation and scalability of model-building procedures, allowing to easily process large-scale image databases, and the evolution of computing technologies such as graphical processing units (GPUs) to speed up the solving process of biophysical simulations.

## Additional file

Additional file 1:
**Table of reviewed 3D cardiac computational models.** **Table S1.** is a wide summary table that lists the main features included in the sixty 3D cardiac computational models reviewed in this work as well as the material and related methods. It also provides information about the purpose of each model and its online availability. The sixty reviewed models appear in the table sorted by chronological order. Authors strongly encourage the readers to download Additional file 1 in order to get all the information extracted from this review and to be able to compare different models in a straightforward and easy manner. **Table S2**. shows the meaning of the acronyms and abbreviations used to encode the information contained in Table S1. Additional file 1 also includes some text explaining what kind of information is provided by each column in Table S1 [185-187].

## Abbreviations

2D: bi-dimensional
3D: three-dimensional
AP: Action potential
APD: Action potential duration
BSPM: Body surface potential maps
BZ: Border zone
CAD: Computer-aided design
CCS: Cardiac conduction system
CRT: Cardiac resynchronisation therapy
CT: Computed tomography
CV: Conduction velocity
DE-MRI: Delayed enhancement-magnetic resonance imaging
DTI: Diffusion tensor imaging
DT-MRI: Diffusion tensor-magnetic resonance imaging
EAMs: Electroanatomical maps
ECG: Electrocardiogram
EM: Electromechanical
EP: Electrophysiology or electrophysiological
FEM: Finite element method
GPUs: Graphical processing units
HH: Hodgkin and Huxley
LBBB: Left bundle branch block
LV: Left ventricle
MRI: Magnetic resonance imaging
MS-CT: Multi slice-computed tomography
ODEs: Ordinary differential equations
PCA: Principal components analysis
PDEs: Partial differential equations
PDM: Point distribution model
PMJs: Purkinje-myocardium junctions
RFA: Radiofrequency ablation
RV: Right ventricle
SD: Standard deviation
VT: Ventricular tachycardia
VV: Interventricular.
