# Supplementary material for: Three-dimensional cardiac computational modelling: methods, features and applications
Source: Biomed Eng Online. 2015 Apr 17;14:35. doi: 10.1186/s12938-015-0033-5 (PMC4424572; doi:10.1186/s12938-015-0033-5)
Supplement: Additional file 1: — Table of reviewed 3D cardiac computational models. Table S1. is a wide summary table that lists the main features included in the sixty 3D cardiac computational models reviewed in this work as well as the material and related methods. It also provides information about the purpose of each model and its online availability. The sixty reviewed models appear in the table sorted by chronological order. Authors strongly encourage the readers to download Additional file 1 in order to get all the information extracted from this review and to be able to compare different models in a straightforward and easy manner. Table S2. shows the meaning of the acronyms and abbreviations used to encode the information contained in Table S1. Additional file 1 also includes some text explaining what kind of information is provided by each column in Table S1 [185-187]. [file 12938_2015_33_MOESM1_ESM.pdf]

MODEL	*Kind of model*	*Species (anatomy)*	*Model extension*	*Anatomical information*	*Segmentation method*	*Meshing*	*Fibre orientation*	*CCS*	*Endocardium detail*	*Other features*	*Model purpose*	*Online availability*
**Koushanpour & Collings, 1966 [1]**	Geom	Rat, cat, turtle	LV	ExpM							MA	
**Okajima *et al.* 1968 [8]**	Anat	Human	BV	HS(3mm)	MD	vFEMh(3mm)		HPS, AK(Dog)			EP	
**Ghista & Sandler, 1969 [2]**	Geom	Human	LV	VLV							MA	
**Janz & Grimm, 1972 [3]**	Geom	Rat	LV, FAn	HS		vFEMh(198el)					MA	
**Horan *et al.* 1978 [9]**	Anat	Human	LV	HS(18)	MD	vFEMh(1675el,3.2mm)		AP, Dur			EP	
**Miller & Geselowitz, 1978 [10]**	Anat	Human	BV	HS(16,4.64mm)	MD	CA(4000,3.75mm)					EP	
**Vinson *et al.* 1979 [185]**	Anat	Human	LV	VLV	M	vFEMh(36el)					MA	
**Van den Broek, 1980 [4]**	Geom	Rabbit	LV	ExpM, ML			RBM				MA	
**Aoki *et al.* 1987 [14]**	Anat	Human	BV	pHS(7)	M	CA(50e3,1.5mm)		HPS, Dur			EP	
**Thakor & Eisenman, 1989 [15]**	Anat	Dog	BV	pHS(1.5mm)	M	vFEMh(1473el)		Purk, AK			EP	
**Nielsen *et al.* 1991 [12]**	Anat	Dog	BV	ExpM		vFEM-H(24el,41n)	DExp(SH)		Pap		MA, EP	AMDB
**Creswell *et al.* 1992 [26]**	IM	Dog	BV	iMRI(11,5mm)	M	vFEMh(x)						
**Lorange & Gulrajani, 1993 [27]**	IM	Human	WH	eCT(132)		CA(25e4,1mm)	Ventricles	HPS, 1120-PMJ			EP	
**Colli Franzone *et al.* 1998 [5]**	Geom		LV			vFEMh(x)	RBM	AP, Dur			EP	
**Vetter & McCulloch, 1998 [11]**	Anat	Rabbit	BV	HS(2-3mm)		vFEMx	DExp(SH)				EM	1
**Siregar *et al.* 1998 [18]**	CAD	Human	WH, GCV	ML		CA(x)	Lit	AVN, HPS, Dur			EP	
**Yamaki *et al.* 1999 [186]**	Anat	Human	WH			CA(50e3,1.5mm)		SAN, AVN, HPS, Dur		ISC	EP	
**Freudenberg *et al.* 2000 [16]**	Anat	Human	WH	pHS-VHP(1mm)	SA2D: CTS	CA(x)		SAN, AVN, HPS, AK(Human)	Pap		EP	
**Harrild & Henriquez, 2000 [19]**	CAD	Human	A	ML		vFEMh(25e4el,0.55mm)		Atr	Pec, FO		EP	
**Winslow *et al.* 2000 [28]**	IM	Rabbit	BV	eMRI(128,469 μm)	SA2D: SN	vFDG(x)	DTI(SH)				EP	
**Blanc *et al.* 2001 [22]**	Geom	Human	A	ML		sFEMt(250e3n,0.2mm)					EP	
**Zemlin *et al.* 2001 [23]**	Anat	Human	A	pHS-VHP(1mm)	SA2D: CTS	sFEMt(6e5el,0.28mm)	DExp(DH)	Atr	Pec, CT		EP	
**Schulte *et al.* 2001 [49]**	IM, PS, Def	Human	BV, FAn	iMRI(5mm)	M	sFEM-H					MBS	
**Virag *et al.* 2002 [29]**	IM, LD	Human	A	xMRI	M	sFEMt(50-400e3n)			FO		EP	
**Lorenzo-Valdés *et al.* 2002 [42]**	At	Human	BV, EpLV	14-iCine-MRI(8-10)	M						MBS-TCM	
**Frangi *et al.* 2002 [51]**	At + Stat	Human	BV, EpLV	14-iMRI(10mm)	M	sFEMt(x)					MBS	
**Kerckhoffs *et al.* 2003 [6]**	Geom		LV			EP - vFEMh (10e3el,11e3n,2mm) Mec - vFEMh(108el,3.2e3n)	RBM	AP(4), Dur			EM	
**Stevens *et al.* 2003 [13]**	Anat	Pig	BV	ExpM		vFEM-H(79el)	DExp(SH)				Mec	
**Sermesant *et al.* 2003 [76]**	IM, Def	Dog	BV	eDT-MRI	SA2D: CM	vFEMt(10e3el,2e3n)	DTI(DH)			LAR	MBS	
**Lötjönen *et al.* 2004 [55]**	At + Stat	Human	WH, EpV	25-iMRI(6-7mm)	M	sFEMt(x)			Pap		MBS	
**Helm *et al.* 2005 [30]**	IM	Dog	BV	eDT-MRI(0.8mm)	SA2D: SN	vFEM-H(24el)	DTI(SH)					
**Haddad *et al.* 2005 [37]**	IM, PS, Dyn	Human	WH, GCV, pCT	iMRI(2mm), iCine-MRI(7mm)	WH - M pCT - SA2D	sFEMt(x)				MCC		
**Perperidis *et al.* 2005 [54]**	At, Dyn	Human	BV, EpLV	26-iCine-MRI(10mm)	M + Reg	sFEMt(x)			Pap	MCC	O	
**Bodin & Kuz'min, 2006 [20]**	CAD	Human	WH, OEp, GCV	O		sFEMt(x)					EP	
**Seemann *et al.* 2006 [24]**	Anat	Human	A	pHS-VHP	SA2D: RG, SN	vFEMh(1.58e6el)		SAN, Atr	Pec, FO		EP	
**Appleton *et al.* 2006 [38]**	IM, PS, Dyn	Human	BV	iCine-MRI(9)	A2D: CM, SN	CA(40e3)		HPS		MCC(20)	EP	
**Yang *et al.* 2006 [40]**	IM, PS	Human	WH, OE	iMS-CT(1mm)	SA2D: SN, LS							
**Burton *et al.* 2006 [44]**	IM, HD	Rabbit	BV, fCT	eMRI(24.4μm), pHS-St(10μm)	MRI - SA2D: CM pHS - A2D: CTS	vFEM	IM3D(SH)		Pap, TC	LT	EP	

Table S1 (part 1). The main features of the sixty reviewed 3D cardiac computational models and the methods used for its development

MODEL	Kind of model	Species (anatomy)	Model extension	Anatomical information	Segmentation method	Meshing	Fibre orientation	CCS	Endocardium detail	Other features	Model purpose	Online availability
**Sermesant *et al.* 2006 [7]**	Geom, Def	Human	BV			vFEMt(20e3el,4e3n)	DTI(DH)			LAR	Mec, MBS	
**Lorenz & von Berg, 2006 [56]**	At + Stat	Human	WH, EpLV, GCV, pCT	27-iMS-CT(0.5mm)	SA3D: Fit	sFEMt(x)					MBS	AMDB
**Sermesant *et al.* 2006 [160]**	(1) Anat, Def, Dyn (2) IM, Def, Dyn	Dog	BV	(1) ExpM (2) eDT-MRI(x)	SA2D: CM	vFEMt(40e3el,7e3n)	(1) Lit(Dog) (2) DTI(DH)	AP, Dur		LAR	EP, Mec, MBS-TCM	
**Trunk *et al.* 2007 [17]**	Anat	Human	WH, GCV, pCT	pHS-VHP	M	Vox(1mm)					O	
**Ordas *et al.* 2007 [43]**	At	Human	WH, pCT	100-iMS-CT(2mm)	A3D: Reg	vFEMt(0.5mm)	RBM	Atr, HPS, AK(Human)		LAR, TH	EP	AMDB
**Peyrat *et al.* 2007 [77]**	At	Dog	WH	9-eMRI			DTI(SH)					2, AMDB
**Arevalo *et al.* 2008 [31]**	IM	Dog	WH	eMRI(0.8mm)	SA2D: CM, RG, LS	vFEMt (29e6el,5e6n,0.4mm)	DTI(SH)		Pap, TC	ISC	EP	
**Plotkowiak *et al.* 2008 [32]**	IM	Rabbit	BV	eMRI(24.4µm)	A2D: LS	vFEMt(3.7e6el,83e4n)			Pap		EP	
**Ecabert *et al.* 2008 [47]**	At + Def, Stat	Human	WH, EpLV, GCV	28-iMS-CT (0.67-3mm)	SA3D: Fit	sFEMt (14.7e3el,7.3e3n,2.5-5mm)					MBS	AMDB
**Ruiz-Villa *et al.* 2009 [21]**	CAD	Human	A	ML		vFEMh(51e3el,1e5n)	Lit(Human)	Atr	Pec, FO	LAD	EP	
**Niederer *et al.* 2009 [39]**	IM, PS	Human	BV	iMRI(x)	M	vFEM-H(112el,183n)	Lit(Dog)				Mec	
**Plank *et al.* 2009 [45]**	IM, HD	(1) Rabbit (2) Rat	BV	eMRI(24.4µm), pHS-St(10µm)	MRI - SA2D: LS, CM pHS-A3D: Reg(MRI)	(1) vFEMt (24e6el,4.3e6n,125µm)	(1) RBM (2) DTI(SH)		Pap, TC	LAR	EP	
**Vadakkumpadan *et al.* 2009 [53]**	IM, HD	Rabbit	BV, fCT	eMRI(24.5µm)	SA2D: CM, RG, LS	vFEMx(31e6el,50µm)	DTI(SH)	Purk, hrMRI	Pap, TC	ISC	EP	
**Heidenreich *et al.* 2010 [33]**	IM	Human	BV	eDT-MRI(0.8mm)	M[11]	vFEMh (1.3e6el,1.4e6n,0.4mm)	DTI(SH)	HPS		RVD, TH	EP	
**Romero *et al.* 2010 [41]**	IM, PS	Human	BV	iMS-CT(x)	A3D: MBS	vFEMt(15-21e6el,2.5-3.5e6n,>0.5mm)	RBM	Purk, Dur		LVH, LVD (3 models)	EP	
**Bishop *et al.* 2010 [46]**	IM, HD	Rabbit	BV, fCT, FAn	eMRI(24.4µm)	SA2D: LS	vFEMt (41.5e6el,7e6n,125µm)	RBM		Pap, TC, CTen	LAR	EP	3
**Wenk *et al.* 2010 [50]**	IM	Sheep	LV, FAn	iMRI(x), pHD		vFEMh(x)	Lit(Dog)		Pap, CTen	ISC	Mec	
**Gurev *et al.* 2011 [34]**	IM	Dog, Human (3 models)	BV	eMRI(x)	SA2D: CM, RG, LS	EP - vFEMx (1.7e6el,1.4e6n) Mec - vFEM-H (172el,356n)	DTI(SH)	AP, Dur			EM	
**Deng *et al.* 2012 [35]**	IM	Human	WH	eCT(531,0.33mm)	SA2D: CM, RG		DExp(SH)	SAN, Atr, AVN, HPS, AK(Human)	Pec, FO		EP	
**Zhao *et al.* 2013 [25]**	Anat, HD	Sheep	A	pHS(50µm)	SA2D: CM, RG	vFDG(0.1mm)	IM3D(SH)	SAN, Atr	Pec, CT		EP	
**Aslanidi *et al.* 2013 [36]**	IM, HD	Dog	A	eMicro-CT(36µm)	SA2D		eMicro-CT(SH)	AVN, Atr	Pec			
**Hoogendoorn *et al.* 2013 [52]**	At + Stat, Dyn	Human	WH, EpLV, GCV, pCT	138-iMS-CT(2mm)	A3D: Reg	sFEMt(16e3n)				LAR, MCC(15)	MBS-TCM, O	4

Table S1 (part 2). The main features of the sixty reviewed 3D cardiac computational models and the methods used for its development

AMDB: Anatomical Model Database (see ref. [187]). http://amdb.isd.kcl.ac.uk:8080/AMDBWebInt/

1: http://cmrg.ucsd.edu/

2: https://team.inria.fr/asclepios/data/

3: https://chaste.cs.ox.ac.uk/trac/browser/data/public

4: http://www.cistib.org/cistib_shf/index.php/translation/downloads

Kind of model	
**Geom**	Geometrical shape-based model (ellipsoid-based)
**SAnat**	Simple anatomical model
**IM**	Image-based model
**PS**	Patient-specific model
**CAD**	CAD model
**Def**	Deformable model
**Stat**	Statistical cardiac model
**At**	Cardiac atlas
**Dyn**	Dynamic model
**HD**	High level of anatomical detail
**LD**	Low level of anatomical detail
Model extension	
**LV**	Left ventricle model
**BV**	Bi-ventricle model
**A**	Bi-atrial model
**WH**	Whole heart model
**GCV**	Great cardiac vessels
**pCT**	Part of coronary tree
**fCT**	Full coronary tree
**OE**	Only endocardium
**OEp**	Only epicardium
**EpLV**	Only epicardium for LV
**EpV**	Only epicardium for ventricles
**FAn**	Fibrous annulus of atrio-ventricular valves
Anatomical information	
**ExpM**	Experimental measurements taken on explanted hearts
**ML**	Measurements taken from the literature
**pHD**	Pictures of heart dissections
**HS(n,mm)**	Histo-anatomical slices. n: number of slices. mm: slice thickness.
**pHS(n,mm)**	Pictures of histo-anatomical slices
**pHS-St(n,mm)**	Pictures of histo-anatomical slices with special staining
**eMRI(n,mm)**	*Ex-vivo* MRI
**iMRI(n,mm)**	*In-vivo* MRI
**N-iMRI(n,mm)**	*In-vivo* MRI. N: population size (for atlases)
**eCT(n,mm)**	*Ex-vivo* CT
**iCT(n,mm)**	*In-vivo* CT
**N-iCT(n,mm)**	*In-vivo* CT
**VLV**	*In-vivo* ventriculograhy of the LV (cine-angio-cardiography)
Segmentation method	
**MD**	Manually drawn
**M**	Manual segmentation
**SA2D**	Semi-automatic 2D (slice by slice), with some manual interaction
**A2D**	Automatic 2D, without any manual interaction
**SA3D**	Semi-automatic 3D segmentation, with some manual interaction
**A3D**	Automatic 3D segmentation, without any manual interaction
**CM**	Classical image processing methods (*thresholing, edge detection, morphological op., etc.*)
**CTS**	Colour-thresholding segmentation
**RG**	Region growing
**SN**	Snakes
**LS**	Level sets
**MBS**	Model-based segmentation
**Reg**	Registration with a previously manually segmented image
**Fit**	Fitting an initial mesh to the target image

General	
**x**	*Feature not included* or *Method not reported*
**O**	Other options
Meshing	
**CA(n,mm)**	Cellular automaton. n: number of cells. mm: spatial resolution.
**sFEM-t(el,n,mm)**	Surface finite element mesh with triangular elements. el: number of elements. n: number of nodes. mm: spatial resolution.
**vFEM-h(el,n,mm)**	Volumetric finite element mesh with hexahedral elements
**vFEM-t(el,n,mm)**	Volumetric finite element mesh with tetrahedral elements
**sFEM-H(el,n,mm)**	Surface finite el. mesh based on cubic Hermite basis functions
**vFEM-H(el,n,mm)**	Volumetric finite el. mesh based on cubic Hermite basis functions
**vFDG(mm)**	Volumetric finite difference grid. mm: spatial resolution.
**Vox(mm)**	Voxels-based volumetric model (*not FEM*). mm: spatial resolution.
Fibre orientation	
**RBM**	By a rule-based method based on Streeter's findings
**DTI**	From *ex-vivo* DTI images
**SH**	From the same heart used for the anatomical reconstruction
**DH**	From a different heart than used for the anatomical reconstruction
**DExp**	From direct experimental measurements
**Lit(sp)**	Taken from the literature. sp: species
**IM3D**	From the volumetric image assembled from histological slices
Cardiac conduction system	
**AP(n)**	CCS emulated by activation points on the endocardial surfaces. n: number of activations points
**Dur**	From the activation maps obtained by Durrer *et al.* 1970
**HPS**	His-Purkinje fibres
**AVN**	AV (atrio-ventricular) node
**SAN**	SN (sino-atrial) node
**Purk**	Only Purkinje fibres
**AK(sp)**	From the anatomical knowledge. sp: species
**N-PMJ**	Purkinje-muscle junctions. N: number of PMJs
**Atr**	Atrial conduction bundles: crista terminals, Bachmann's bundle and pectinate muscles
**hrMRI**	Free-running Purkinje fibres from high-resolution *ex-vivo* MRI
Endocardium detail	
**Pap**	Papillary muscles
**TC**	Trabeculae carnae
**Pec**	Pectinate muscles
**CT**	Crista terminalis
**FO**	Fossa ovalis
**CTen**	Chordae tendineae
Other features	
**LAR**	Labelling of anatomical regions
**TH**	Electrophysiological transmural heterogeneity in ventricular wall
**RVH**	RV hypertrophy
**LVH**	LV hypertrophy
**LVD**	LV dilation
**LAD**	Left atrium dilation
**ISC**	Infarct-derived ischemic scar in LV, including core and border zone
**MCC(n)**	Motion due to the cardiac cycle. n: number of phases
**LT**	Labelling of tissues (histological information)
Model purpose	
**MA**	Mechanical analysis
**EP**	Simulation of cardiac electrophysiology
**EM**	Simulation of cardiac electro-mechanics
**Mec**	Simulation of cardiac mechanics
**MBS**	Model-based segmentation
**MBS-TCM**	Model-based segmentation with tracking of cardiac motion

Table S2. List of acronyms used in the table of reviewed 3D cardiac computational models (Table S1)

Content of Table S1

First column in Table S1, named “*Kind of model*”, corresponds to a proposed classification based on the level of anatomical realism achieved by the model and the method used for the 3D reconstruction of the cardiac anatomy.

Second column, called “*Species (anatomy)*”, specifies the animal species whose anatomy is modelled.

Under the heading “*Model extension*” the cardiac chambers and structures included in each model are detailed.

Next two columns provide information about the source of the “*Anatomical information*” and the “*Segmentation method*” (for image-based models) used to build each model, respectively.

The column labelled as “*Meshing*” shows the approach used to generate the 3D computational model from the reconstructed cardiac geometry and, if reported, some details such as mesh resolution.

“*Fibre orientation*” and “*CCS*” (cardiac conduction system) columns report whether or not these features are included in the model and, if so, the approach used to include them.

“*Endocardium detail*” column gives information about the level of anatomical detail achieved in the reconstructed endocardial surfaces, both in ventricles and atria.

Next column, named “*Other features*”, collects miscellaneous information, such as the inclusion in the model of ischaemic scars, some kind of anatomical variation, labelling of interesting anatomical regions, etc.

“*Model purpose*” column specifies the final application for which each model was originally developed.

The last column, “*Online availability*”, reports whether the model is available online, providing the link if so.
